# Different factors drive the assembly of pine and *Panax notoginseng*-associated microbiomes in *Panax notoginseng*-pine agroforestry systems

**DOI:** 10.3389/fmicb.2022.1018989

**Published:** 2022-11-14

**Authors:** Weijia Jia, Shu Wang, Xiahong He, Xiaoyan Zhao

**Affiliations:** ^1^College of Landscape Architecture and Horticulture Sciences, Southwest Forestry University, Kunming, China; ^2^Ministry of Education Key Laboratory for Forest Resources Conservation and Utilization in the Southwest Mountains of China, Southwest Forestry University, Kunming, China

**Keywords:** agroforestry system, land use conservation, rhizosphere, endophyte, microbial transmission

## Abstract

Land-use conversion affects the composition and assembly of plant-associated microbiomes, which in turn affects plant growth, development, and ecosystem functioning. However, agroforestry systems, as sustainable land types, have received little attention regarding the dynamics of different plant-associated microbes. In this study, we used high-throughput sequencing technology to analyze the assembly mechanisms and the driving factors of pine- and *Panax notoginseng* (*P.n.*)-associated microbiomes during the conversion of different pine forests (*Pinus kesiya* var. *langbianensis* and *Pinus armandii*) into *P.n.*-pine agroforestry systems. The results showed that the conversion of pure pine forest into *P.n.*-pine agroforestry systems significantly altered the diversity of pine-associated fungi rather than the community structure, and the community structure of *P.n.*-associated fungi rather than the diversity. Additionally, plant-associated fungi were more responsive to land-use change than bacteria. Main effect analysis revealed that compartment rather than genotype was the driving factor of pine- and *P.n.*-associated microbiomes, but *P.n.* cultivation also significantly affected the assembly of pine-associated microbiomes. In addition, there was a transfer of *P.n.* endophytes to pine trees in agroforestry systems and the beneficial microbiomes (Massilia, Marmoricola, Herbaspirillum, etc.) were enlarged in pine roots. Therefore, the diversity of the assembly mechanisms of *P.n.*- and pine-associated microbiomes played an important role in the *P.n.*--pine agroforestry systems and were the basis for the sustainable development of the *P.n.*--pine agroforestry systems.

**Figure:**
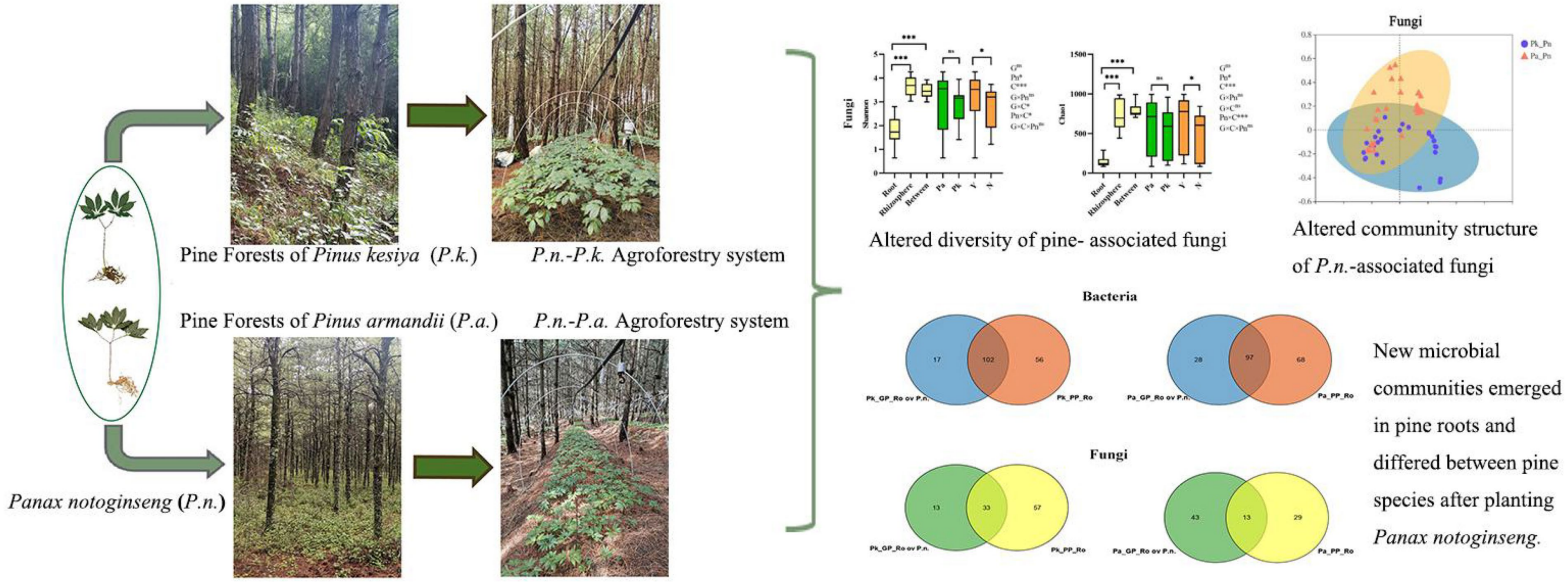
Graphical Abstract

## Highlights

– The conversion of pure pine forests into *Panax notoginseng-*pine agroforestry systems affected plant-associated microbiomes.
– The assembly of Pine- and *Panax notoginseng*-associated microbiomes had different influencing factors.
– Compartment rather than genotype was the driving factor of *Panax notoginseng* and pine-associated microbiomes, but *Panax notoginseng* cultivation also affected the assembly of pine associated microbiomes.
– There was a diffuse spread of *Panax notoginseng* endophytes into the pine roots, and beneficial microbiomes (Massilia, Marmoricola, Herbaspirillum, etc.) increased in pine roots.

## Introduction

Plant-associated microbiomes include endophytes in plant tissue and rhizosphere soil microbiomes, mainly include bacterial and fungal taxa. These microbiomes play an important role in plant development and plant physiological state prediction (Montesino, 2003; Hardoim et al., 2015). Some endophytes have been proven to promote plant growth (Chandra et al., 2018) and accumulate beneficial components (Tiwari et al., 2010; Brader et al., 2014). Especially for medicinal plants, endophytes can regulate the synthesis of key secondary metabolites and increase the content of effective components, such as participating in the transformation and increasing the concentration of ginsenosides in ginseng (Song et al., 2017b; Fu, 2019) and promoting the accumulation of berberine in *Coptis teeta* (Liu et al., 2020). Similarly, many rhizosphere soil microbes (such as PGPB and PGPF) can enhance plant nutrient absorption and utilization (Van Der Heijden et al., 2008; Balsanelli et al., 2019) and enhance crop quality (Verginer et al., 2010; Nasopoulou et al., 2014). They are especially important for the formation of high-quality medicinal plants, such as improving the nutritional element enrichment of *Paris polyphylla* (Zhang H. Z. et al., 2019), and participating in the synthesis of indigo in *Baphicacanthus cusia* and artemisinin in *Artemisia annua* (Zeng et al., 2018; Zhai et al., 2019). Therefore, plant-associated microbiomes have a significant impact on the quality of plants, especially medicinal plants.

The assembly of plant-assiocated microbiomes follows the theory of microbial ecology and is affected by stochastic and deterministic assembly processes (Dini-Andreote et al., 2015). Previous studies have shown that in high microbial diversity communities, stochastic assembly processes are dominant for most cases, while in low microbial diversity communities, deterministic assembly processes are dominant (Kembel, 2009; Xun et al., 2019). And the potential and stability of ecosystems can be predicted by determining their relative contributions (Tilman et al., 1997; Duffy et al., 2017; Galand et al., 2018). In plant–microbe interactions, the assembly of plant-associated microbiomes is affected by specific driving factors, such as genotype (Bulgarelli et al., 2013; Xu et al., 2020), plant tissue (Dong et al., 2018; Alibrandi et al., 2020), soil conditions (Qiao et al., 2017; Long and Yao, 2020) and host-associated environments (Campisano et al., 2017; Cai et al., 2020). Fungi and bacteria are differentially affected due to their physiological and evolutionary differences. Bacteria, such as the rhizosphere bacteria of maize (Ren et al., 2020), the endophytic bacteria of *Fagus sylvatica* (Coince et al., 2014) and rice (Feng et al., 2019), and the root-related bacteria of the medicinal plant *Polygonum cuspidatum* (Zhang et al., 2020), are mainly shaped by environmental variables and soil factors, while fungi, such as the root-related fungus *Helianthus annuus* (Leff et al., 2017) and the rhizosphere fungi *Picea asperata* and *Abies faxoniana* (Li et al., 2021), are more sensitive to soil nutrient contents and host inheritance. In addition, plant interactions are also crucial to the shaping of plant-assiocated microbiomes. For example, mangroves have a potential impact on the colonization of root endophytic bacteria of *Spartina alterniflora* (Hong et al., 2015). However, either interspecific or intraspecific impact of plant interactions, especially the relationship between *Panax notoginseng* (*P.n.*) flora and pine flora, on plant-associated microbes (fungi and bacteria) is scarcely studied. Therefore, the study of plant–microbe–plant interactions is of more significance to the assembly mechanism of plant-related microbes (fungi and bacteria), especially the transmission of plant-related microbes.

Agroforestry, as one of the land use conversion practices, is not only an important factor in the change in microbial composition and structure, but also a sustainable strategy to alleviate the shortage of cultivated land resources and the environmental burden (Anderson and Sinclair, 1993; Montagnini and Nair, 2004). Previous studies on the variation in soil microbial communities under agroforestry management have yielded different results. Several studies have found that the soil microbial diversity of agroforestry systems is richer than that of forests (Edy et al., 2019; Beule et al., 2020), and, some studies have found that soil microbial diversity remains stable, but community composition is significantly altered when forests are converted to agroforestry systems (Banerjee et al., 2016; Wang et al., 2017), while some others have concluded that native forests can maintain microbial richness and diversity better than agroforestry systems (Belay et al., 2020). In addition, agroforestry is also one of the determinants of plant endophytes. For example, some studies have shown that agroforestry increases the diversity of moso bamboo endophytes and significantly alters their community composition (Zhang H. Z. et al., 2019; Zhang X. et al., 2019). Similarly, studies have found that the richness and the colonization rate of arbuscular mycorrhizal fungi of crops increase under agroforestry management (Sousa et al., 2013; Edy et al., 2019). In fact, the changes in endophytic bacteria in crops are closely related to soil microbiomes. Studies have examined those endophytic fungi of trees and crops that are transmitted to the soil (Ingleby et al., 2007). Therefore, the combination of soil microbial changes and plant endophytic changes in agroforestry systems is of great significance for further understanding the dynamics of plant microbes within the systems.

*Panax notoginseng* (*P.n*., Araliaceae) is a precious traditional medicinal herb in China (Yang et al., 2019; Wang et al., 2020). However, under traditional agricultural management, the serious continuous cropping obstacle of *P.n.* results in a shortage of arable land and a decline in yield and quality (Liu et al., 2011; Wang et al., 2019). The *P.n.*-pine agroforestry system, as an organic cultivation strategy for medicinal plants to restore their native habitat, is a necessary approach to ensuring the quality and pharmacological activity of *P. n.* and soil fertility. The success of this organic cultivation depends on the interplay between *P.n*., pine trees, and the environment (Yu and Zhang, 2019; Wu et al., 2021). However, the relationship between plants and microbiomes in the *P.n.*-pine agroforestry systems is still unclear. Therefore, in this study, four land types including pure pine forests (*Pinus kesiya* var. *langbianensis* and *Pinus armandii*) and agroforestry systems (*Pinus kesiya* var. *langbianensis* - *Panax notoginseng* and *Pinus armandii* - *Panax notoginseng*) were targeted to analyze the changes and influencing factors of microbiomes associated with *P.k.*, *P.a.* and *P.n*. using 16S amplicons and fungal ITS sequencing techniques. Based on the experimental design, the following hypotheses were made: (I) *P.n*. cultivation in agroforestry systems can alter pine-related microbiomes; (II) different agroforestry systems (Lancang and Xundian) would drive different assemblies of *P.n*.-related microbiomes; (III) microbial transmission would exist within the agroforestry systems between pine and *P.n*. This studyaim to reveal the characteristics and driving mechanisms of plant microbial variation in the *P.n.*-pine agroforestry systems.

## Materials and methods

### Study site

The research was mainly carried out in the pure forest of *Pinus kesiya* var. *Langbianensis* (*P.k.*) in Lancang Lahu Autonomous County, Pu′er City, Kunming, Yunnan Province (99.82°E, altitude of 1457.39 m, mean annual temperature of 19.2°C, mean annual precipitation of 1008.6 mm) and the pure forest of *Pinus armandii* (*P.a.*) in Dadishui Village, Xundian Hui Autonomous County, Kunming, Yunnan Province (103.21°E, 25.47°N, altitude of 2247.81 m, mean annual temperature of 15.5°C, mean annual precipitation of 1624.0 mm). Both forests are located in the central and western parts of the Yunnan-Guizhou Plateau and are fallow forest stands with a regular plant spacing of about 3 m. The vegetation types are shown in the following table (Supplementary Table S1).

### Experimental design and sample collection

**Figure 1:**
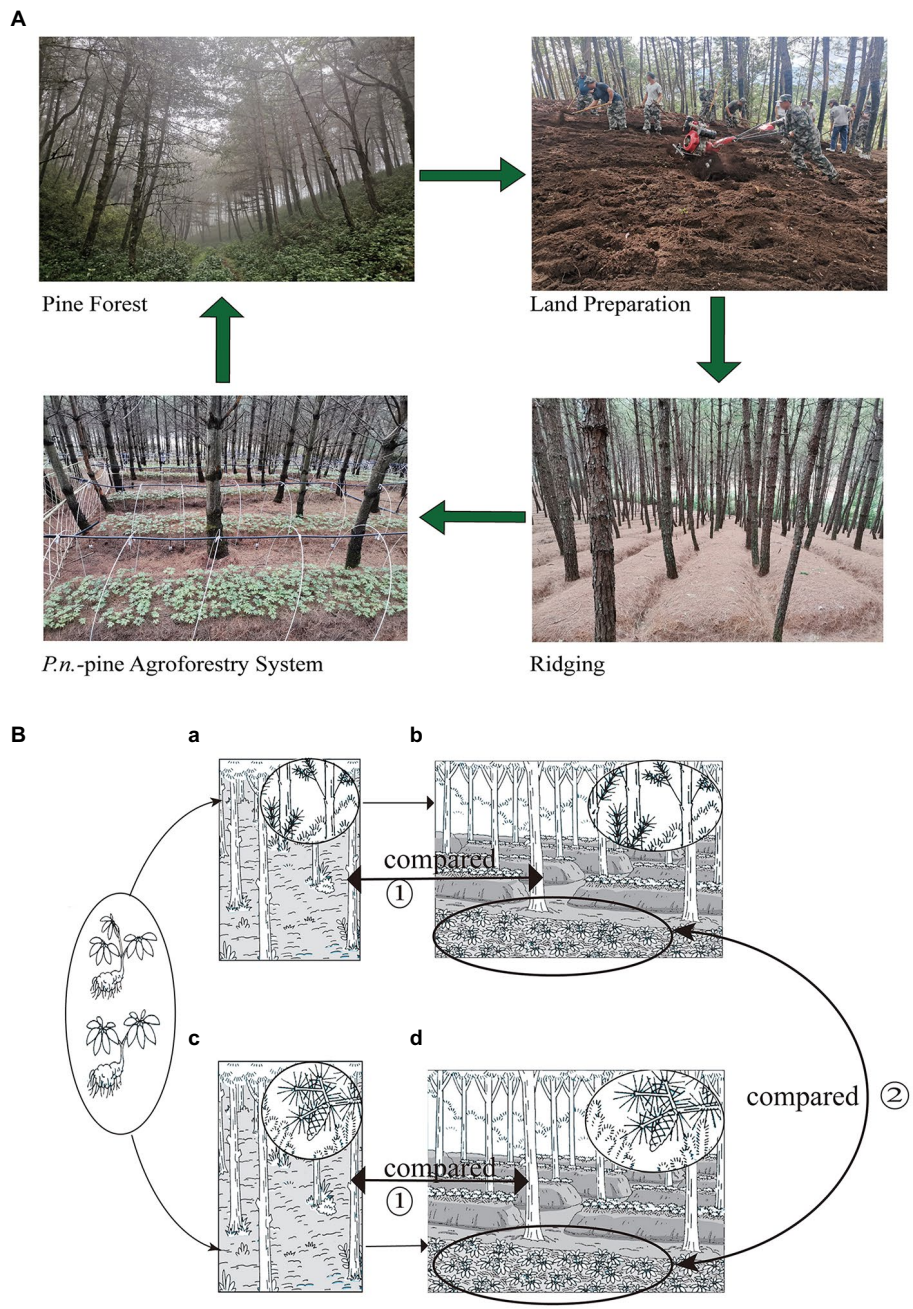
Specific planting process of the *Panax notoginseng* - pine agroforestry systems **(A)** and experiment design **(B)**. Experiment one (①) compared the pure forests of *P.k.* (a) and *P.a.* (c) with the corresponding *P.n.*-pine agroforestry systems (b,d) for the difference of pine-associated microbes. Experiment two (②) compared two *P.n.* -pine agroforestry systems (b,d) for the differentence of *P.n.* -associated microbes.

Forested areas of *P.k.* and *P.a.* were selected as mentioned in 2.1 respectively, for *P.n.* understory cultivation. Before the cultivation., weeds, small shrubs, and dead leaves were removed from the understory planting area. *P.n.* were planted in a ridge along the contour of each forest, with a height/bottom/top size of the ridge of 40 cm x 120 cm x 80 cm. The ridges were covered with 3 cm pine needles to retain water and heat (the specific planting process is shown in Figure 1). *P.n.* seedlings (two genotypes: trifurcated five-leaf and bifurcated seven-leaf) had been obtained after greenhouse cultivation in November 2018 before they were transferred to the forests in November 2019, with a plant spacing of 10 cm × 10 cm. The *P.n.*-pine agroforestry systems were constructed using a typical low-input “eco-agriculture” model, by which the use of pesticides and fertilizers was prohibited and only daily irrigation (a small amount) was provided to avoid drought. In addition, shelters were built during the rain season to prevent flooding.

**Figure 2:**
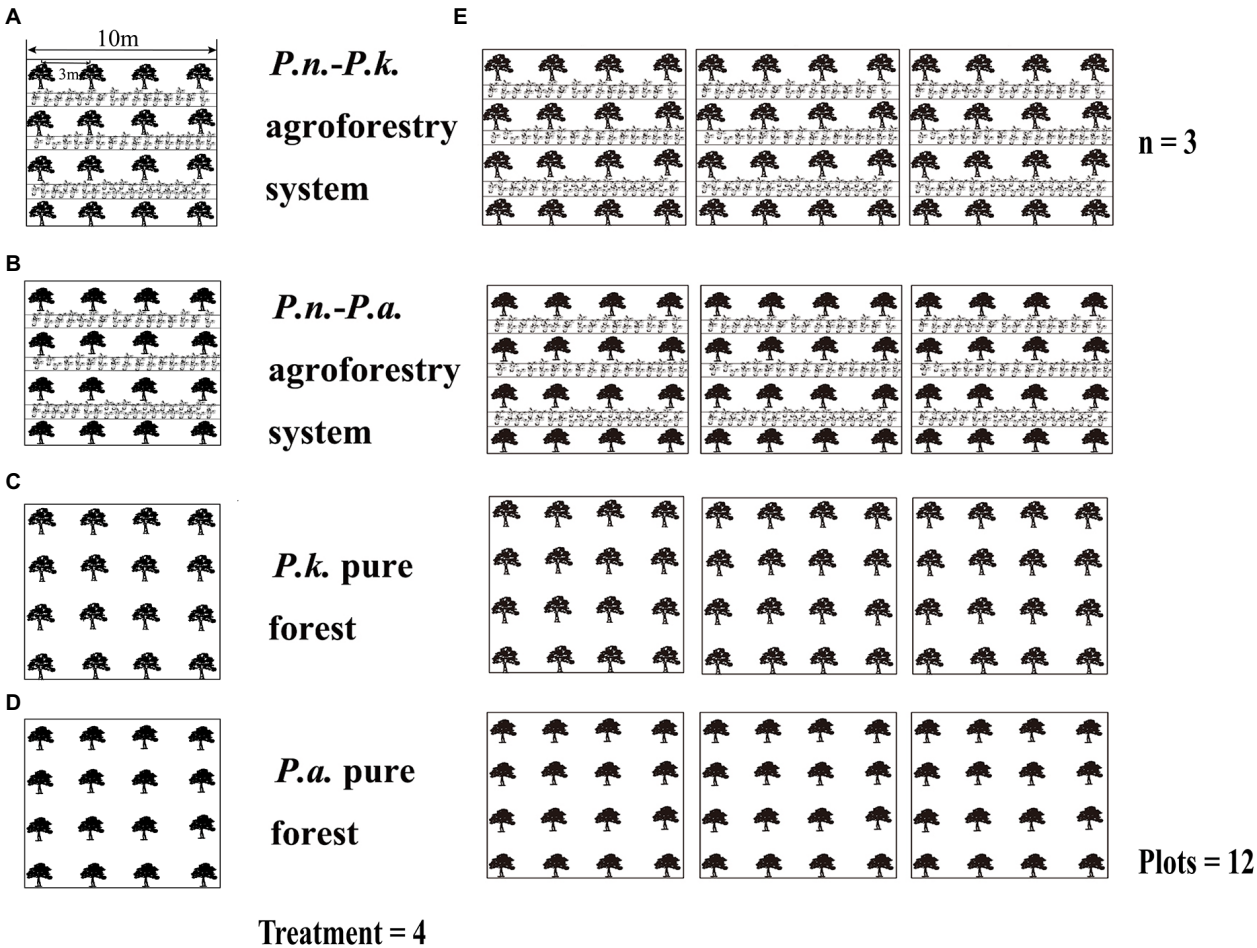
Four types of sampling plots and sampling plot design. [sample plots of *P.n.*-*P.k.* agroforestry system **(A)**, *P.n.*-*P.a.* agroforestry system **(C)**, and two types of pine pure forest **(B,D)** and sample plots design **(E)**].

The experiments were divided into two parts, Experiment One compared pure pine forests with the corresponding *P.n.*-pine agroforestry systems to explore the effect of land conversion on pine-associated microbiomes. Experiment Two compared *P.n.*-pine agroforestry systems with different pine species to explore the effect of different pine species on the assembly of *P.n.*-associated microbiomes (Figure 2). Therefore, four types of sampling plots (10 m × 10 m, plot spacing is more than 5 m) were set up in Lancang (L) and Xundian (X), i.e., *P.n.*-*P.k.* plots, pure *P.k.* forest plots, *P.n.*-*P.a.* plots and pure *P.a.* forest plots, and were replicated three times, totaling 12 plots (Figure 2; the samples within the pure forest plots were noted as PP, and the samples of *P.n.*-pine plots were noted as GP or PG). In Experiment One, pine root samples (named L_GP_Ro, L_PP_Ro, X_GP_Ro, and X_PP_Ro, respectively) and pine rhizosphere soil (named L_GP_Rh, L_PP_Rh, X_GP_Rh, X_ PP_Rh, respectively) in *P.n.*-*P.k.* plots, pure *P.k.* forest plots, *P.n.*-*P.a.* plots and pure *P.a.* forest plots; soil at the locations where the pine trees and the ridges meet (named L_GP_Be, X_GP_Be, respectively) in the *P.n.*-pine plots (*P.k.*, *P.a.*) and bulk soil of pine trees at the corresponding locations (named L_PP_Bu, X_PP_Bu, respectively) in the pure forest plots (*P.k.*, *P.a.*) were collected. In Experiment Two, roots, stems, and leaves of bifurcated seven-leaf and trifurcated five-leaf *P.n.* (named L_SL_Ro, L_SL_St, L_SL_Le, L_FL_Ro, L_FL_St, L_FL_Le, X_SL_Ro, X_SL_St, X_SL_Le, X_FL_Ro, X_FL_St, X_FL_Le, respectively), rhizosphere soil of bifurcated seven-leaf and trifurcated five-leaved *P.n.* (named L_FL_Rh, L_SL_R, X_FL_Rh, X_SL_Rh, respectively) *in P.n.*-pine (*P.k.*, *P.a.*) plots, and bulk soil of *P.n.* (named L_PG_Bu, X_PG_Bu, respectively) *in P.n.*-pine (*P.k.*, *P.a.*) plots and soil at the locations where the pine trees and ridges meet (L_PG_Be/X_GP_Be) *in P.n.*-pine (*P.k.*, *P.a.*) plots were collected.

*P.n.* is planted in the forest for 1 year and then stalked and regrown for another year, so sampling was conducted before the harvest of *P.n.* on November 20, 2020. The collection of plant samples in each sampling plot was divided into two parts: the collection of pine roots and *P.n.* plants. Pine root sampling: Five pine trees more than 50 cm away from the four sides of a sampling plot were randomly selected, and 15–20 young healthy roots (root diameter < 2 mm) of each pine tree were collected and mixed as a plant sample. *P.n.* sampling: Ten *P.n.* Plants of two genotypes with similar growth performance were collected from the ridges adjacent to the selected pine trees in a *P.n.*-pine sampling plot, and the roots, stems, and leaves were separated. Soil sampling adopted the five-point method, which removed plant litter from the soil surface, collecting four soil cores (sampling depth 0–20 cm) at approximately 50 cm from the four corners of the sampling plot, collected one soil core at the center of the sampling plot, and thoroughly mixed them into one soil sample. The rhizosphere soil was the soil immediately attached to the plant roots (0–2 mm from the root surface). The sample size of pure forest plots in this experiment was 18 (3 samples of the individual plot × 2 (two pine species) × 3 replicates), and the sample size of *P.n.*-pine plots was 72 (12 samples of the individual plot × 2 (two agroforestry systems) × 3 replicates), which added up a total of 90. Plant and soil samples were stored in liquid nitrogen and dry ice, respectively, and then immediately transported back to the laboratory and stored at −80°C for further analysis.

### Plant and soil physicochemical properties

The fresh soil samples were passed through a 0.298 mm sieve to determine the physical and chemical properties. The soil water content was measured by drying the soil at 85°C for 48 h to constant weight, and the soil pH and EC were determined by a 5:1 water/soil suspension with a pH meter and a conductivity meter, respectively. Total nitrogen (TN), total phosphorus (TP), NH_4_^+^ − N, and NO_3_^−^−N were measured by a SmartChem200 analyzer using standard methods. TK was analyzed by atomic emission spectrometry on an AA-6300C flame photometer.

After the plants were collected, they were rinsed with pure water, dried with filter papers, and measured for fresh weight. They were then dried at 70° to constant weight, and measured for dry weight and water content. Afterwards, they were milled and 0.5 g of the milled plant samples was digested by Kjeldahl decoction (10 ml 95.5%H_2_SO_4_ and 3%H_2_O_2_), and the digestion solution was used to determine the TN and TP of plants (Lv et al., 2004; Wang et al., 2022) by a SmartChem200 analyzer.

### DNA extraction, PCR amplification, and sequencing

The plant samples for endophyte collection were soaked and rinsed with sterile water, 70% ethanol, and 2.5% NaClO for 1 min, 90 s, and 30 s, respectively. Then sonication procedures were performed twice with phosphate-buffered saline and observed under a scanning electron microscope to ensure that all microbes were removed from the plant surface. DNA extraction from soil samples (0.5 g each) used No, 12888.100 Qiagen DNeasy PowerSoil Kit (MP Biomedicals, Solon, CA, United States) and from 0.5 g of the plant tissue samples (the pine roots and the roots, stems, and leaves of *P.n.*) used the Qiangen DNeasy Plant Kit following the manufacturer’s instructions. The PCR amplification procedure and sequencing process are detailed in the Supplementary material. The bacterial 16S rRNA and fungal ITS gene sequencing was performed on the Illumina MiSeq PE300 platform. We filtered the OTUs assigned to chloroplasts and mitochondria from the OTU table before further analysis. Sequences have been deposited in the National Center for Biotechnology Information Sequence Read Archive under Accession No. PRJNA821648 (16S RNA data) and No. PRJNA821834 (ITS data).

### Statistical analysis

Differences in the physicochemical properties of the plants (*P.n.*, pine roots) and the soil, microbial alpha diversity, and the relative abundance of major phyla/genera in this study were analyzed using various ANOVA methods. For comparisons between two groups, either the one-way Student’s t test (normally distributed variables) or the Mann–Whitney nonparametric test (other variables) was used. For comparisons between multiple groups, the one- to multi-way ANOVA with a *p* value less than 0.05 was considered to be a significant difference. The microbial diversity (Shannon index), richness (Chao1), and evenness (Shannon even index) were selected to characterize the microbial alpha diversity. Alpha diversity was assessed in relation to soil and plant physicochemical properties using Pearson correlation analysis. These analyses were all performed using SPSS 25.0 (IBM, Armonk, NY, United States).

Venn diagrams and stacked bar charts were based on microbial OTU Tables (97% similarity level)were implemented using R (version 3.3.1) to visualize microbial community composition and differences. Microbial beta diversity was calculated in QIIME (1.9.1) based on weighted UniFrac distance. PCoA plots made with the vegan and ggplot2 packages in R were used to visualize differences in community composition. Permutational multivariate analysis of variance (PERMANOVA, 999 permutations calculated) was used to examine the effects of planting *P.n.* (pine species), compartment, genotype, and their interactions effects on the community composition of pine (*P.n.*)-associated microbiomes. Pairings between planted *P.n.* (pine species) and genotypes were also calculated to compare effects between different compartments (bulk, between, rhizosphere, root, stem, leaf for *P.n.* and bulk, rhizosphere, root for pine). Detrended correspondence analysis (DCA) was performed with microbial sample OTU tables (from the first axis of the length of the gradient) to determine RDA/CCA (redundancy analysis/Canonical correspondence analysis) and was used to further assess the effect of plant soil physicochemical properties on bacterial and fungal communities. These were analyzed and plotted using the Vegan package in R.

## Results

### Variations in plant-assoicated microbial richness and diversity

**Figure 3:**
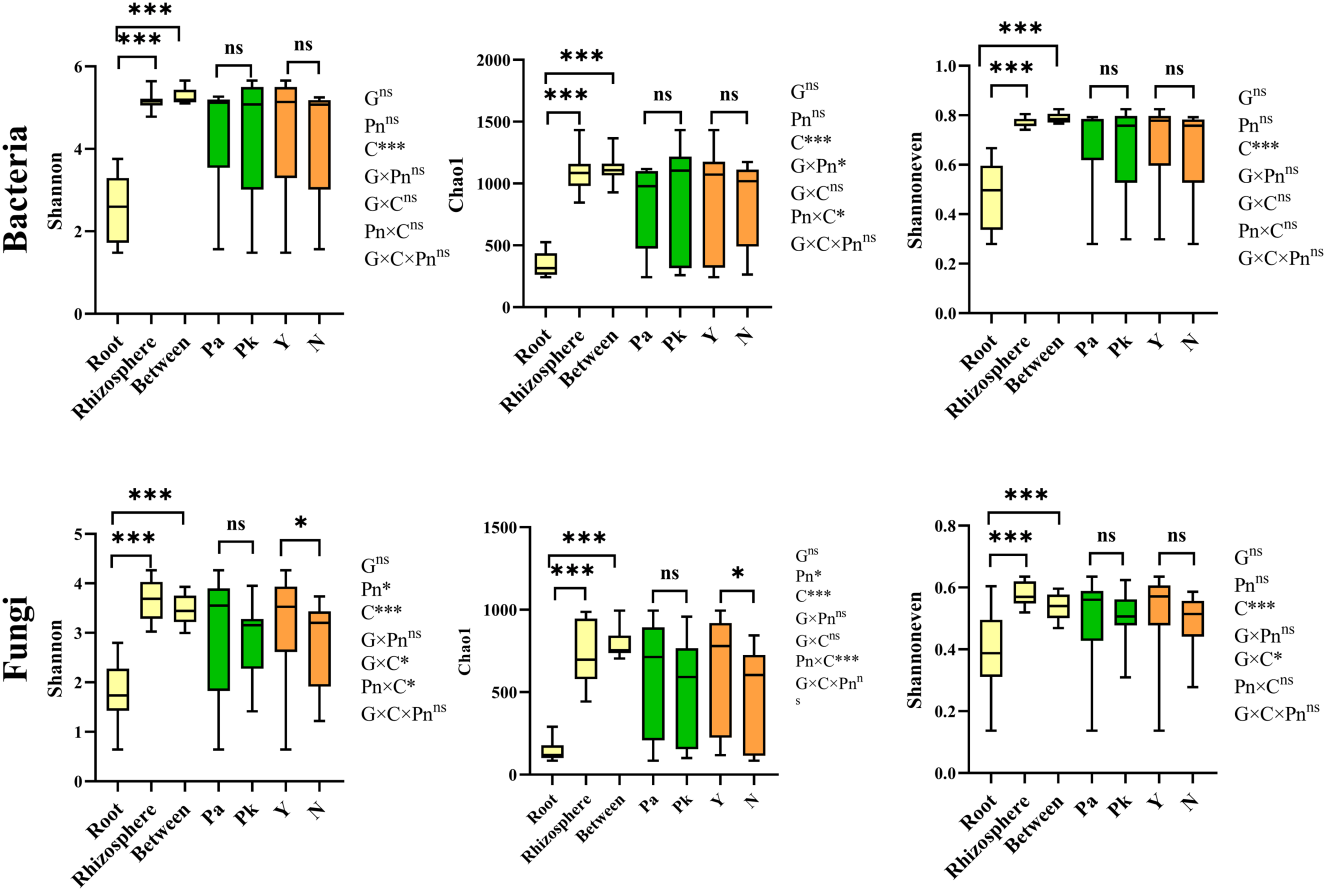
Boxplots of indices of bacterial and fungal alpha diversity of microbes in samples from different compartments (between (bulk) and rhizosphere soils, and root of pine trees), genotype and planting *P.n.* or not. Different letters showed significant difference (*p* < 0.05). The Significance of effects of genotype (G), compartment (C) and planting *P.n.* or not (Pn) and their interactions on microbial diversity was evaluated by multi-way ANOVA. *, p < 0.0 5, and ***, *p* < 0.001, ns, no significance.

Compared to pine forests, the α-diversity of pine-associated fungi in agroforestry systems increased, while there was no significant change in bacteria. Main effect analysis showed that compartment and *P.n.* cultivation rather than pine genotype significantly affected the α-diversity (Chao1 and Shannon index and Shannoneven index) of pine-associated microbiomes (Figure 3). In general, the α-diversity of pine-associated microbes showed a decreasing gradient from the soil (bulk and rhizosphere) to the roots. In addition, significant genotype and compartment (G × C) interactions were found in α-diversity of pine-associated fungi but not bacteria. The interaction between planting *P.n.* and compartment (Pn × C) increased the fungal α-diversity in the rhizosphere of pine, while it had no significant effect on the intraroot and bulk soil (Figure 3; Supplementary Table S2). Significant (G × Pn) interactionsexsited in bacteria (rhizosphere and bulk soil) but not in fungi. The indicators that were most closely related to the microbial diversity of the rhizosphere soil of pine were total nitrogen, pH and soil water content. However, the correlations between root endophytes (*P.k*., *P.a.*) and environmental indicators were not significant, except for the correlation between root endophytes and root nitrogen content (Supplementary Table S3).

**Figure 4:**
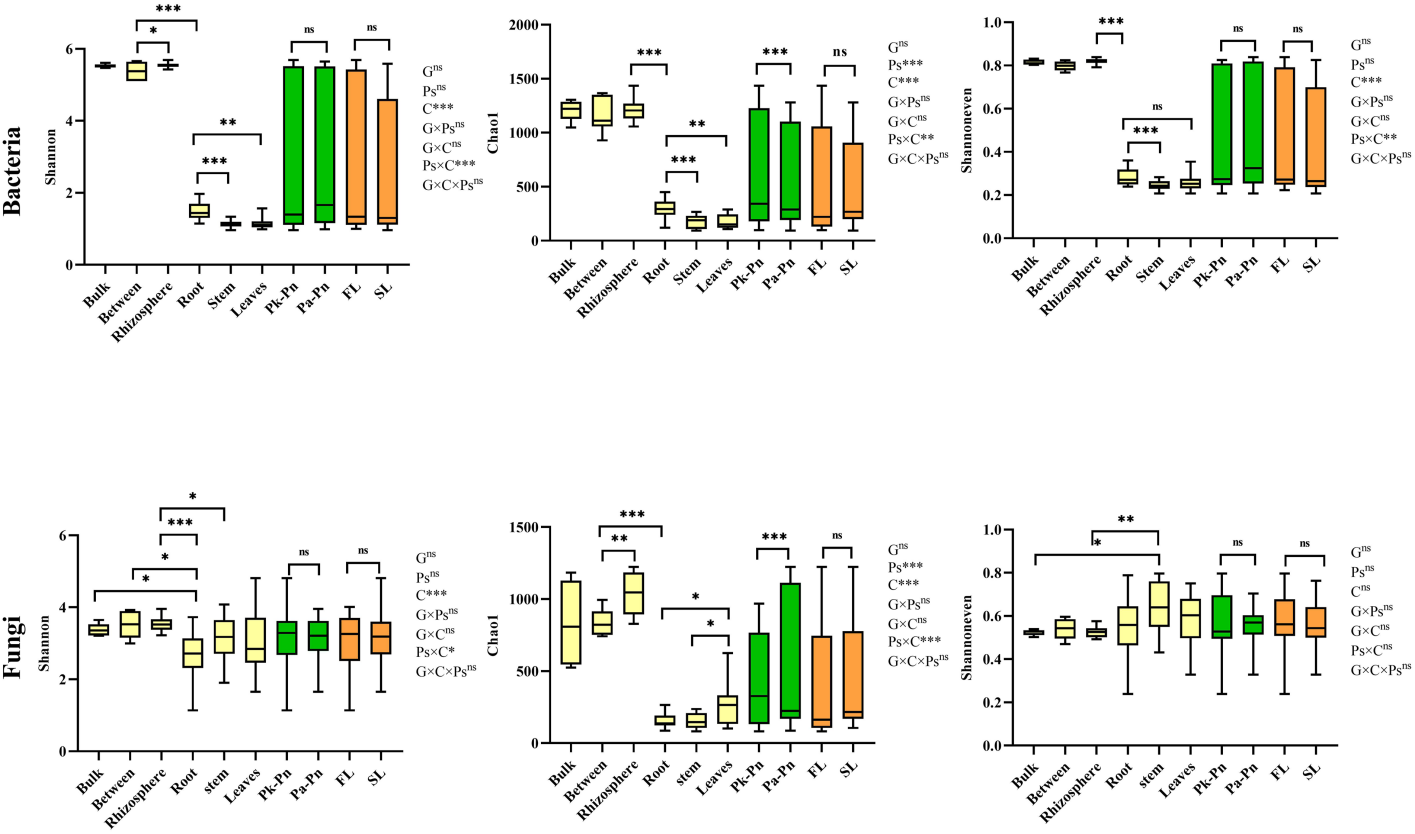
Boxplots of indices of bacterial and fungal alpha diversity of microbes in samples from different compartments (bulk, between, rhizosphere soils, and roots, stems, leaves of *P.n.*), genotype and pine trees species. Different letters showed significant difference (p < 0.05). The Significance of effects of genotype (G), compartment (C) and pine trees species (Ps) and their interactions on microbial diversity was evaluated by multiway ANOVA. *, *p* < 0.0 5, **, *p* < 0.01, ***, *p* < 0.001, ns, no significance.

In agroforestry systems, different pine tree species had no significant effect on the α-diversity of *P.n.*associated microbiomes (bacteria and fungi). Main effect analysis showed that compartment, rather than genotype or pine species, significantly affected the α-diversity of *P.n.-* associated microbiomes (Figure 4). In general, the α-diversity of *P.n.*-associated microbes exhibited a decreasing gradient from the soil (bulk and rhizosphere) to intraplant. As for endophytes, endophytic bacteria were the most abundant in roots, and endophytic fungi were the most abundant in leaves. The interaction effect (Pn × C) between planting *P.n.* and compartment was reflected by the species richness (Chao1) in the rhizosphere soil and leaves of *P.n.* but not in its roots and stems (Supplementary Table S4). Among the morphological and physiological indicators of plants, fresh weight was most closely related to microbiomes (Supplementary Table S5) and soil nitrate nitrogen was more closely related to the α-diversity of *P.n.*-associated microbes. In addition, bacterial α-diversity was also significantly related to the total phosphorus of the soil, and fungal α-diversity was closely related to the TN and EC of the soil.

### Changes in the composition of plant-related microbial communities

**Figure 5:**
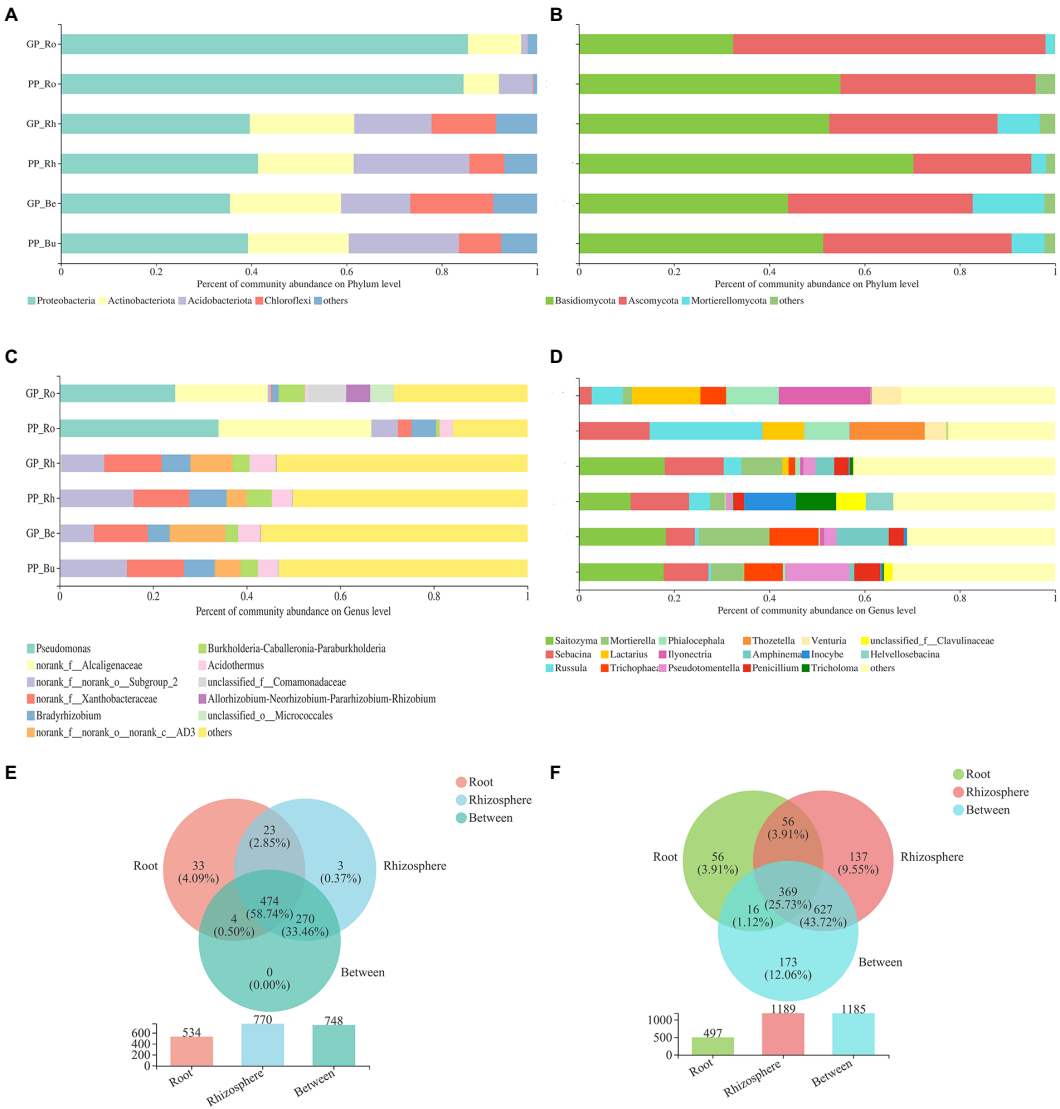
The relative abundance of the most abundant bacterial **(A,C)** and fungal **(B,D)** communities of pine at the phylum and genus level (means, n = 6), Venn diagram showing the number of shared and unique bacterial **(E)** and fungal **(F)** communities at the OTU level.

**Figure 6:**
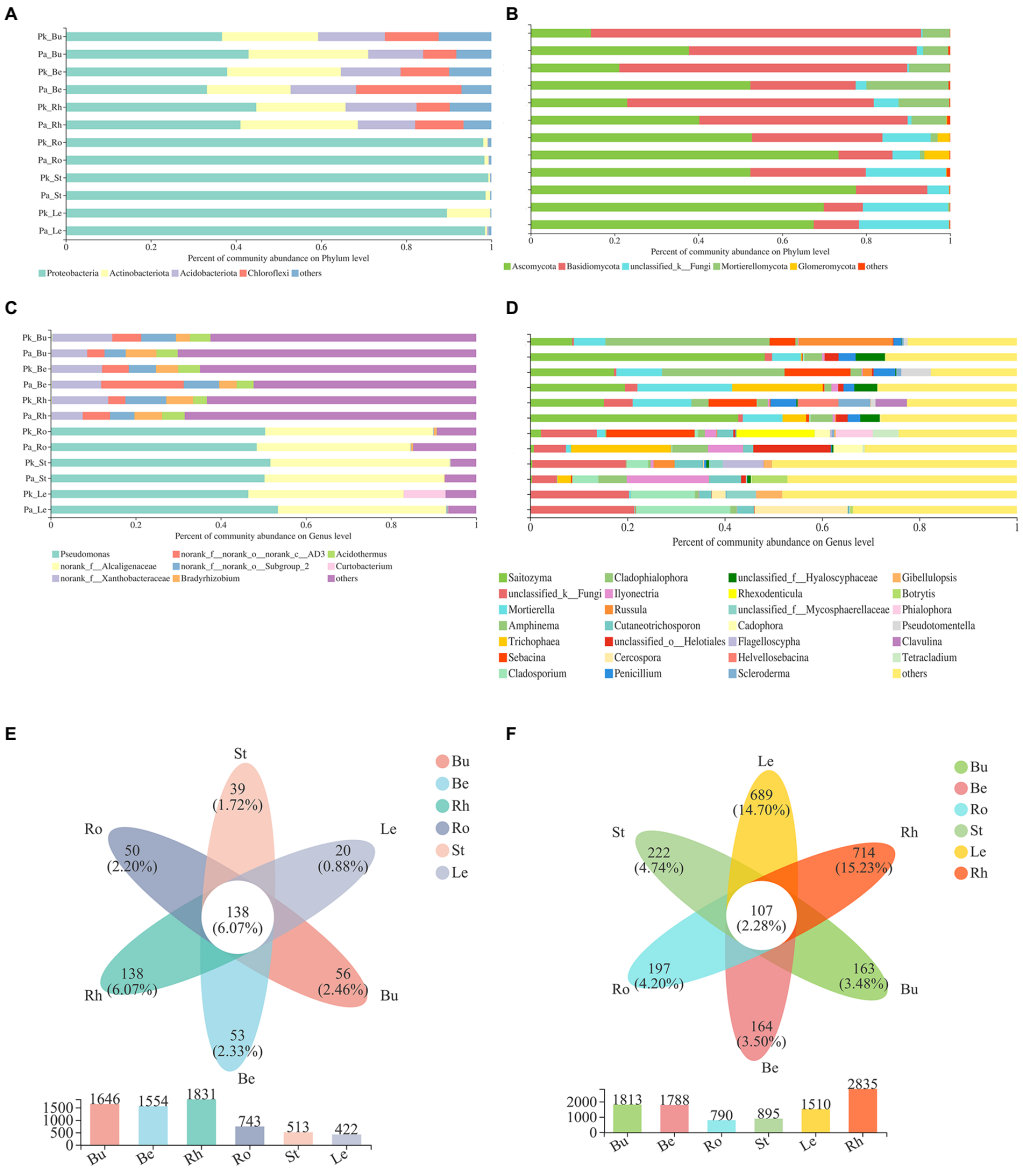
The relative abundance of the most abundant bacterial **(A,C)** and fungal **(B,D)** communities of *P.n.* at the phylum and genus level (means, n = 3), Venn diagram showing the number of shared and unique bacterial **(e)** and fungal **(f)** communities at the OTU level.

Land conversion did not change the community composition of pine and *P.n.*-associated microbiomes (Figures 5, 6).

The dominant phyla of pine-associated bacteria (bulk soil, rhizosphere, root of *P.k.* and *P.a.*) were Proteobacteria, Actinobacteria, Acidobacteria, and Chloroflexi (Figure 5). The main groups of bacteria in roots were similar to those in the soil, but the proportion of Proteobacteria and Actinobacteria in pine roots was larger (more than 90%). The fungal community of pine (bulk soil, rhizosphere, root) was mainly composed of Basidiomycota, Ascomycota, and Mortierella. Land conversion increased the proportion of Ascomycota and Mortierella, and decreased that of Basidiomycota.

At the genus level, the endophytic bacteria of pine roots were mainly composed of Pseudomonas and bacterial genera in the family Alcaligenes, which accounted for more than 40% of bacteria. The rhizosphere bacteria were mainly composed of the genera in the families of Xanthobacteraceae, Bradyrhizobium, Burkholderia-Caballeronia-Paraburkholderia, Acidobacter and genera in the phylum of Chloroflexi, which accounted for 45% above. The endophytic fungi of pine were dominated by Russula, Sabacina, Thozetella, and Lactarius. Soil fungi were dominated by Saitozuma, Sebacina, and Mortierella, accounting for 30%.

In all sequenced samples, the majority of the pine root endophytic microbes were also existed in the soil (rhizosphere and bulk soil), including bacteria (93.82%) and fungi (88.73%), and the number of OTUs of the roots shared by the rhizosphere soil was higher (93.07% of bacteria and 85.5% of fungi) compared with the bulk soil. This result indicated that most of the root endophytic microbes were recovered from the soil environment. Land conversion. Increased the proportion of OTUs specific to pine roots, and the proportion of OTUs specific to fungi was greater than that of bacteria (bacteria increased by 2.2%, fungi increased by 6.84%).

The main composition of *P.n.*-associated soil bacteria was largely similar to that of pine-associated soil bacteria. Proteobacteria (more than 90%) dominated the endophytic bacteria (roots, stems, leaves) of *P.n.* The fungal communities of *P.n.* (soil and root) were mainly composed of Basidiomycota and Ascomycota (accounting for more than 80%). Basidiomycota dominated in the soil, and Ascomycota dominated in the endosphere of *P.n.*

At the genus level, soil bacteria were dominated by the genera in the family Xanthobacteraceae, genera in the phyla Chloroflexi, Bradyrhizobium and Acidothermus, and endophytic bacteria of *P.n.* are dominated by Pseudomonas and the genera in the family Alcaligenaceae. Soil fungi were dominated by Saitozyma, Cladophialophora, Mortierella, and unclassified fungi. Unclassified fungi accounted for the largest proportion of endophytic fungi of *P.n.* A Venn diagram showed that the root endophytic microbial OTUs of *P.n.* overlapped with those of the rhizosphere the most (fungi 28.48%, bacteria 47.99%), while leaf endophytic fungi had more unique OTUs (45.63%).

### The assembly of pine-related microbes and *P.n.*-related microbes is driven by different factors

#### Pine-associated microbial community assembly

**Figure 7:**
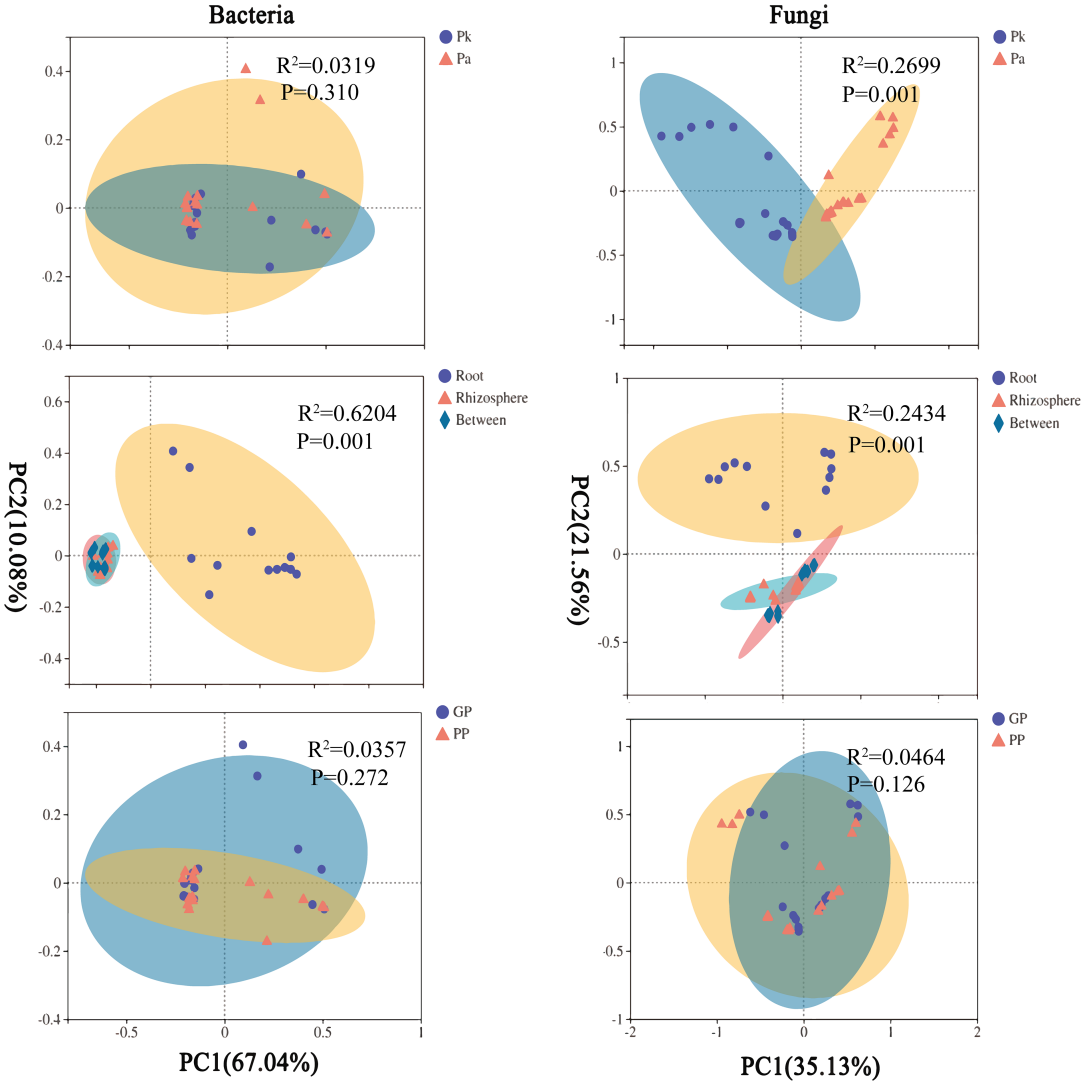
Principal co-ordinates analysis (PCoA) plots based on the weighted UniFrac distance (WUF) of pine-asscioated microbes. Pk, samples of *Pinus kesiya* var. *Langbianensis,* Pa, samples of *Pinus armandii* Franch., GP, *P.n.*-pine agroforestry systems, PP, pure pine forests.

There were no significant changes in the community structure of pine microbiomes (bacteria, fungi) in agroforestry systems compared to pure forests (Figure 7). PERMANOVA showed that compartment significantly affected the community structure of pine microbiomes, and the pine genotype significantly affected the community structure of pine fungi, and planting *P.n.* had limited effects (Supplementary Table S6).

PCoA showed that soil microbes and pine root endophytes were clustered into two groups along principal coordinate 1, indicating that the recruitment of pine endophytes was distinctive. However, both rhizosphere fungi and bulk soil bacteria had a noted distinction between land use types (*P.n.*-pine agroforestry systems vs. pure pine forests), indicating that planting *P.n.* was one of the sources of the β-diversity of the rhizosphere fungal and bulk soil bacterial communities. In addition, the fungal composition in pine roots was only affected by genotype.

**Figure 8:**
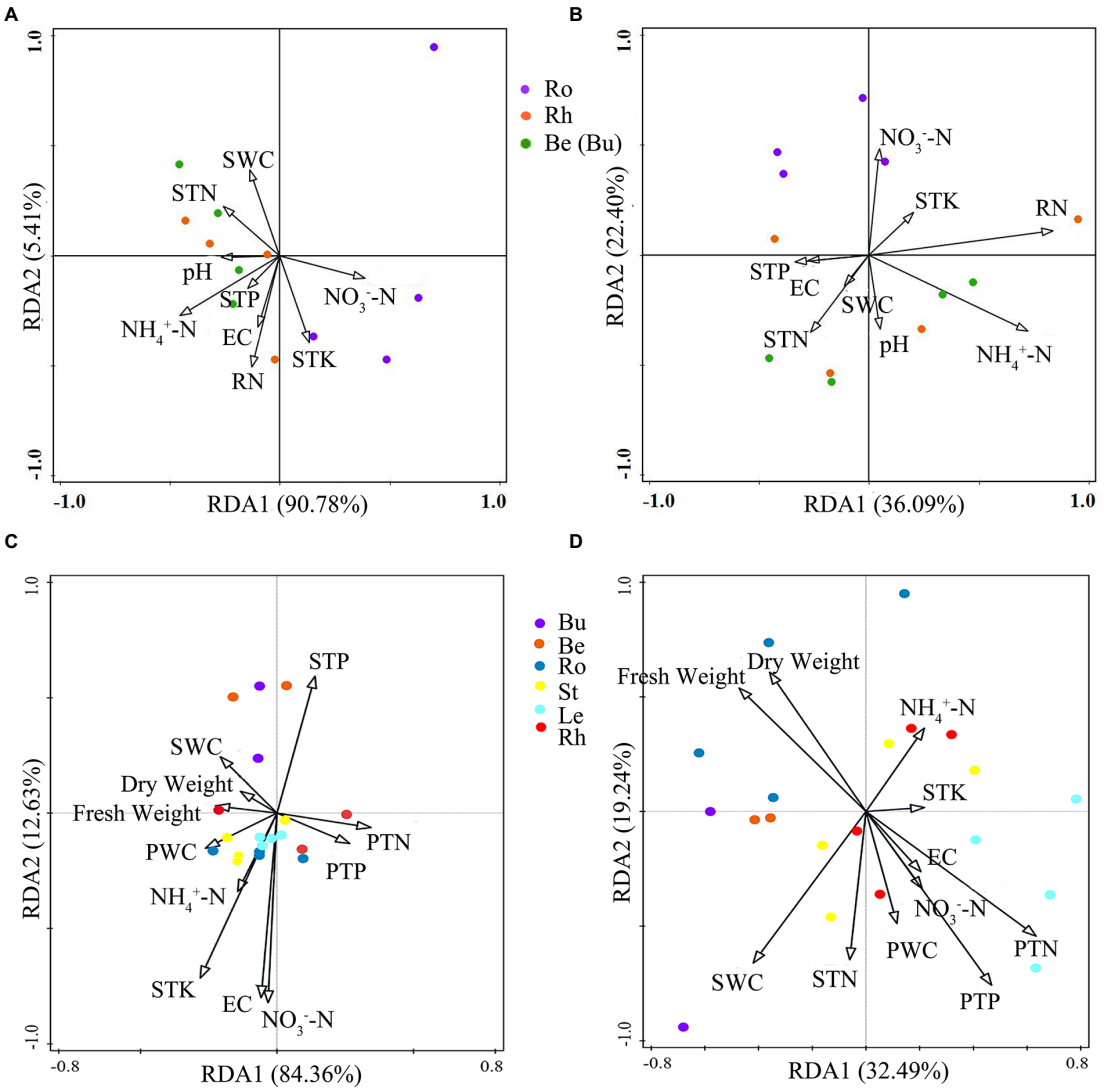
The RDA analysis plots showing the relationship between environmental factors and pine associated bacterial **(A)** and fungal **(B)** communties at the OTU level and the relationship between environmental factors and *P.n.-*associated bacterial **(C)** and fungal communties **(D)** at the OTU level. Soil traits: SWC, soil water content, STN, soil total nitrogen, STP, soil total phosphorous, STK, soil total potassium, NH_4_^+^-N, soil ammonium nitrogen, NO_3_^−^-N, soil nitrate nitrogen, EC, soil conductivity. Plant traits: RN, Root nitrogen, Dry weight, plant dry weight, Fresh weight, plant fresh weight, PWC, plant water content, PTP, plant total phosphorous, PTN, plant total nitrogen.

When determining the effects of biotic and abiotic factors on community composition, the longest lengths for the DCA (detrended correspondence analysis) on the 16S rDNA and ITS datasets were 1.47 and 3.23, respectively. Therefore, RDA was chosen for the analysis of bacterial and fungal communities. Overall, bacterial RDA1 and RDA2 (total explanatory variance: 96.19%) explained more variance in community composition than fungi (total explanatory variance: 58.49%). RDA indicated that ammonium nitrogen, soil water content and total potassium were more correlated with bacterial community composition (Figure 8) and soil water content, nitrate nitrogen and root nitrogen were more correlated with to fungal community composition.

#### Assembly of *P.n.*-related microbial communities

**Figure 9:**
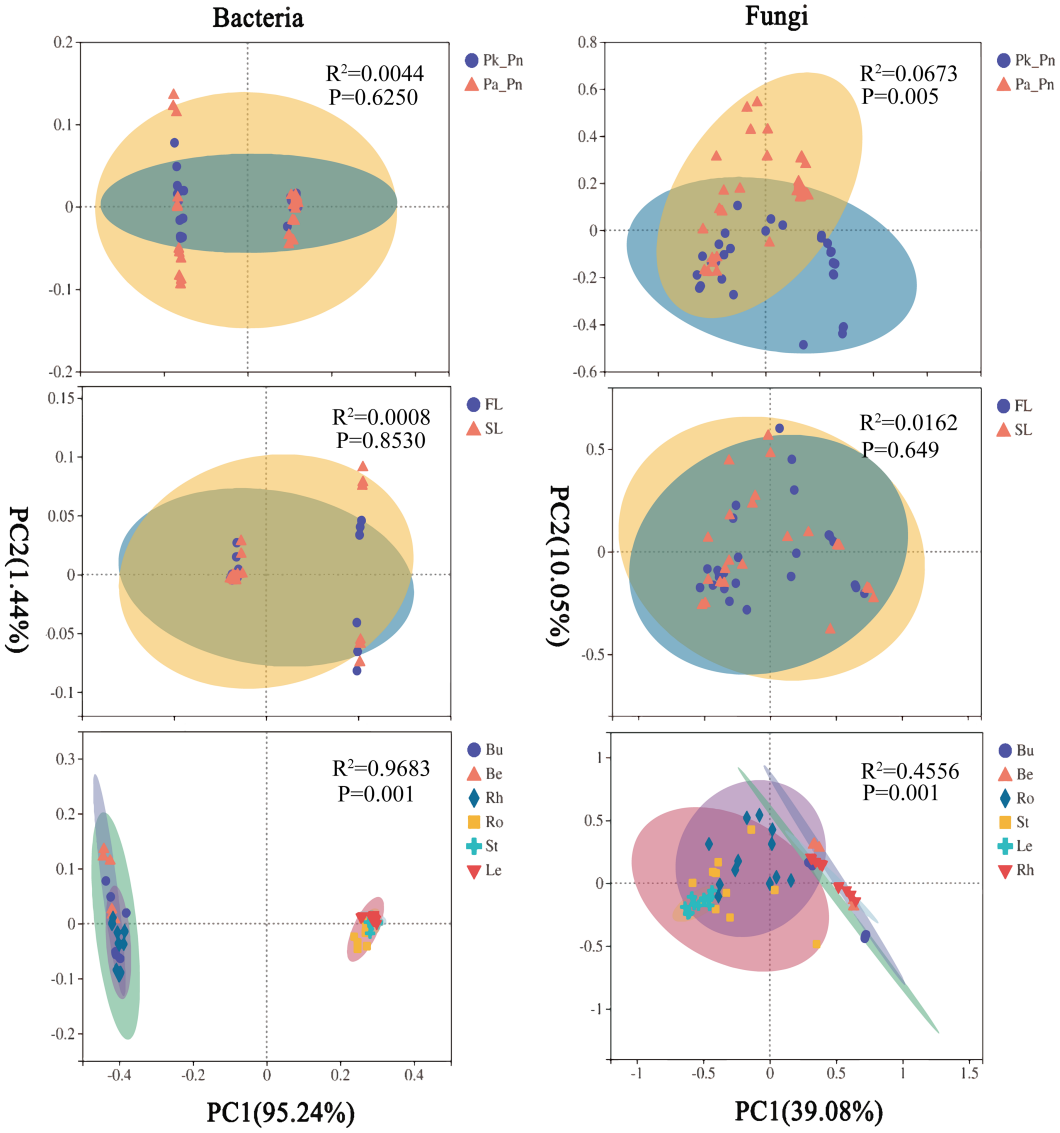
PCoA plots based on the WUF of *P.n.*-associated microbes. Pk_Pn, samples of *P.n.* under *P.n.*-*P.k.* agroforestry system, Pa_Pn, samples of *P.n.* under *P.n.*-*P.a.* agroforestry system, FL, *P.n.* of three palmately compound leaves and five leaflets, SL, *P.n.* of two palmately compound leaves and seven leaflets.

The community structure of *P.n.* fungi was significantly altered in the two agroforestry systems with different pine species (Figure 9). PERMANOVA showed that compartment and pine species, rather than P.n. genotype, significantly affected the community structure of *P.n.*-associated microbiomes (Supplementary Table S7).

PCoA showed that soil microbes and plant endophytes were grouped separately along the principal coordinate 1, indicating that the recruitment of *P.n.* endophytes was specific. In addition, the response of *P.n.*-associated fungi to pine species were different among compartments, and those in the rhizosphere and the root were the most significant (Supplementary Figures S1, S2).

The longest gradients for the DCA of the 16S rDNA and ITS datasets were 1.32 and 2.04, respectively. Therefore, RDA was chosen for the analysis of the microbial communities. Overall, RDA1 and RDA2 of bacteria (total explanatory variance: 96.99%) explained more variance in community composition than fungi (total explanatory variance: 51.73%). RDA showed that the indicators with greater correlations with bacterial community composition were total soil potassium and plant water content (Figure 8), and, plant TN and soil water content were more closely related to fungal composition.

### Partial overlap and differential microbial analysis of pine roots and *P.n* roots in different *P.n.*-pine agroforestry systems

**Figure 10:**
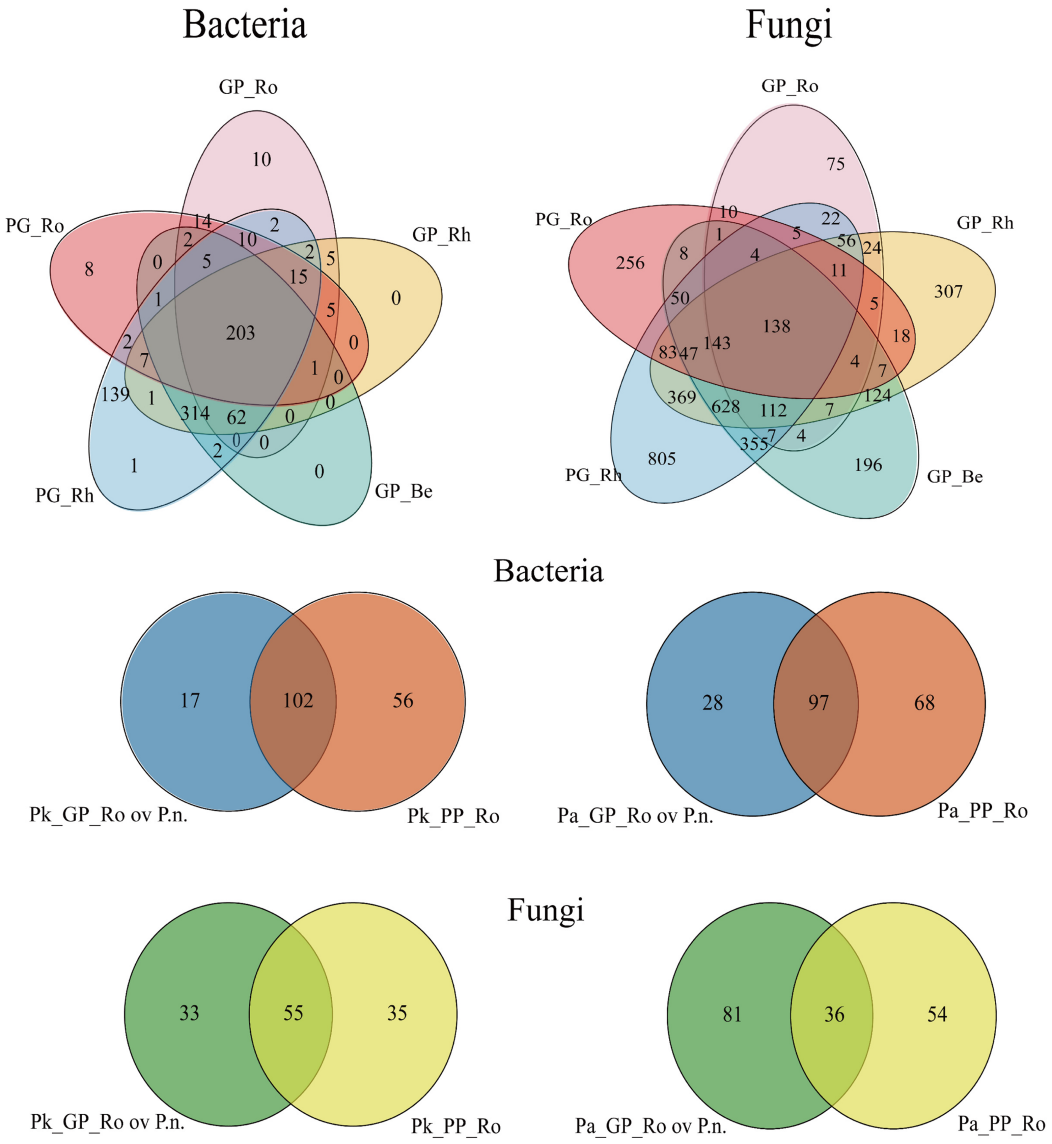
Venn diagram showing the overlap and possible transmission of colonies in the *P.n*.-pine agroforestry system. (Pk/Pa) GP_Ro, the pine root microbes of *P.n.*-(*P.k.*/*P.a*) agroforestry system, (Pk/Pa) GP_Rh, the rhizosphere microbes of *P.n.*-(*P.k.*/*P.a*) agroforestry system, GP_Be, the middle of the planting *P.n.* and adjoining pine tree, PG_Rh, the *P.n.* rhizosphere microbes, PG_Ro, the endosphyte of *P.n.* root. Pk(Pa)_GP_Ro ov *P.n.*, Overlap of the endophytic microbes of *P.k.* (*P.a.*) roots with the endophyte of *P.n.*

There were variations in microbial taxa within pine roots in agroforestry systems compared to pure forests, and the taxa originated from endophytic bacteria of *P.n.* (Figure 10; Supplementary Figure S3). Fungal taxa varied more than bacteria in the rhizosphere of pine and bacterial taxa varied more than fungi in pine roots (Supplementary Figures S4–S7).

In the roots of *P.k.*, the bacteria increased by 23 genera and decreased by 48 genera; the fungi increased by 48 genera and decreased by 31 genera. In the root of *P.a.*, the bacteria increased by 30 genera and decreased by 66 genera, the fungi increased by 82 new genera and decreased by 27 genera. Interestingly, 73.9% of the new bacterial genera and 68.75% of the new fungal genera in the roots of *P.k.*, and 93.3% of the new bacteria and 93.9% of the new fungi in the root of *P.a.* overlapped with the endophytes of *Panax notoginseng* (roots, stems and leaves).

Compared with the pure forests, the microbial changes among each compartment of the pine trees in the *P.n.*-pine agroforestry systems, are shown in the figure (Supplementary Figures S5–S8). The proportions of Acidobacteriales and Acidimicrobiia in the *P.k.* roots were significantly decreased, Biastococcus was significantly increased. However, in the rhizosphere bacteria, the proportion of Acidobacteriaceae was significantly decreased, and norank_c_AD3 and Acidothermus were significantly increased. For the *P.k.* rhizosphere fungi, the proportion of Amphinema and Cenococcum increased significantly, and Tricholoma, Clavulinaceae, and Helvellosebacina genera decreased significantly. Correspondingly, after planting *P.n.*, the proportions of Rhodanobacter and Methylovirgula were significantly increased for endosphere bacteria in the *P.a.* roots. In *P.a.* rhizosphere bacteria, the genera norank_o_TK10 and ADurb_Bin063_1 were significantly increased, and Mycobacterium, Granulicella, and Flavobacterium were significantly decreased. In *P.a.* root fungi, Venturia was significantly reduced. However, the proportions of the Mortierella, Thelephoraceae and Talaromyces genera increased significantly, while those of the Inocybe, Sagenomella, and Tomentella genera decreased significantly in the *P.a.* rhizosphere fungi.

In agroforestry systems with different pine species, the *P.n.*-associated fungi responded to a greater degree than bacteria, and rhizosphere microbes responded to a greater degree than endophytes (roots, stems, leaves of *P.n.*), while stem endophytes were the most stable (Supplementary Figures S8, S9).

Under *P.n.*-pine agroforestry systems of different pine species, Acidothermus, Conexibacter, Rhodanobacter, etc., changed significantlyamong the *P.n.* rhizosphere bacteria, Paraburkholderia, Rhodanobacter MND1, etc., were significantly changed among the *P.n.* root bacteria, Alorhizobium, Pararhizobium and Tardiphaga were significantly changed among the *P.n.* stems bacteria, and Soilbacter and Patulibacter were significantlychanged among the *P.n.* leaf bacteria.

The rhizosphere fungi of *P.n.* changed significantly in the genera Saitomyza and Cladophialophora. In the *P.n.* roots, the genera Trichophaea, Paraglomerales, Sebacina, Phialophora, etc., changed significantly. The unclassified_fungi of Teratosphaeriaceae, Aureobasidium, Papiliotrema, etc., changed significantly in the *P.n.* stems. In the *P.n.* leaves, Aureobasidium, Lapidomyces and Genolevuria changed significantly.

## Discussion

Plant-associated microbiomes influence the health and yield of crops and the functioning of agroforestry ecosystems. The community diversity and composition of plant-associated microbiomes are important indicators of the stability of agroforestry ecosystems. Additionally, tissue type (Dong et al., 2018; Alibrandi et al., 2020), plant introduction (Zhang X. et al., 2019; Beule et al., 2020), plant genotype (Bonito et al., 2014; David et al., 2016), and soil conditions (Qiao et al., 2017; Long and Yao, 2020) are important factors for the assembly of plant-associated microbiomes. In this study, a systematic investigation of the variation and assembly of plant-associated microbiomes under the *P.n.*-pine agroforestry systems has revealed differences in the microbial driving factors associated with *P.n.* and pine.

### Compartment, cultivation of *P.n.*, and pine genotype drive the diversity and community structure of pine-related microbiomes

Compared to pure forests, the α-diversity of pine-associated fungi in the agroforestry systems showed an increasing trend, while bacteria did not change significantly. In addition, there were no significant changes in community structure (β-diversity) and community composition (bacteria and fungi). This is consistent with the results of *Manihot glaziovii*-*Gliricidia sepium* agroforestry system (Sousa et al., 2013). Main effects analysis showed that compartment, *P.n.* cultivation and pine genotype significantly affected the diversity of pine-related microbiomes (α-diversity and β-diversity). The effect of compartment on plant microbial diversity has been demonstrated by many studies (Dong et al., 2018; Alibrandi et al., 2020), and we have found that compartment is the most important factor in the microbial assembly of pine trees in agroforestry systems. Microbial diversity of different compartments (root, rhizosphere, bulk) of pine trees showed that the soil (bulk, rhizosphere) microbes were greater than endophytes (root), which is similar to the results obtained in previous studies on poplar systems (Gottel et al., 2011). This suggested a hierarchical filtering effect (Chen et al., 2016) on the assembly of pine root-associated microbiomes. The root epidermis constituted a natural barrier that creates a filtering effect on the microbiomes that spread to the plant (Bulgarelli et al., 2013). Additionally, the critical influence of compartment might arise from the complex interactions of microbiomes and the ecological differentiation due to the different proportions of substrate and genotype drivers further leading to the formation of different structures of microbiomes in different compartments of pine (Bulgarelli et al., 2013). Planting *P.n.* increased the diversity of fungi associated with pine trees but had no significant effect on bacteria (associated with pine).

As the second important factor, *P.n.* cultivation significantly affected the α-diversity of pine fungi, but not the community structure (bacteria, fungi). Land use conservation, such as forest conservation in the cacao agroforestry system, can significantly affect fungal diversity but not bacterial diversity (Edy et al., 2019). Previous studies have shown that the effect of land conservation on fungi is mainly due to variations in soil organic matter content and plant root vigor (Beule et al., 2020). The introduction of *P.n.* and factors such as tillage and covering with plant litter during the cultivation may have changed the soil chemical and physical properties, thereby affecting the fungal diversity. In addition, there was very little effect of *P.n.* cultivation on the community structure of pine-associated microbiomes, which is different from the result that the invasive plant *Spartina alterniflora* (*S.a.*) significantly changed the root-related microbiomes of *Kandelia obovata* (Hong et al., 2015). This may be because *S.a.* as an invasive plant, was in competition with *K.o.* for nitrogen sources. Although *P.n.* replaced the original herbaceous plants in the pine forests, the nitrogen demand of the whole forest system did not increase, and at the same time, the coveringwith plant litter during the cultivation of *P.n.* also provided a certain nitrogen for *P.n*.

Pine genotype did not have a significant effect on the α-diversity of pine-associated microbiomes (bacteria and fungi), but significantly changed the community structure of fungi (associated with pine). The finding differed from some previous studies which have found a significant effect of host genotype on root-associated bacteria (Bonito et al., 2014), probably because both *P.k.* and *P.a.* belong to the family Pinus spp. and their difference at the taxonomic level is slight. In addition, the differences in fungal community structures may because pine trees, as typical trophic species with ectomycorrhizal symbiosis, are often symbiotic with different fungi and are largely influenced by genotype (Peršoh, 2013).

### Compartment and pine species rather than *P.n.* genotype drive the changes in *P.n.*-associated microbiomes

In the agroforestry systems, different pine species did not significantly affect the α-diversity of *P.n.*-associated microbiomes (bacteria and fungi) but significantly altered the fungal community structure, which is partially consistent with the results obtained in poplar-based alley cropping systems (Beule and Karlovsky, 2021). Main effects analysis showed that compartment and pine species rather than *P.n.* genotype significantly affected the diversity of *P.n.* related microbiomes (α-diversity and β-diversity). Compartments significantly affected the α-diversity of *P.n.*-associated microbiomes, which is consistent with the result for pine trees in this study, suggesting that compartment is an important influencing factors of plant-associated microbial diversity in both herbaceous and woody species. The α-diversity in different compartments of *P.n.* showed that soil microbiomes were greater than endophytes, and endophytes showed *P.n.* root bacteria were more abundant and leaf fungi were more abundant. Moreover, the hierarchical filtering effect of plants was significant in *P.n.*-associated bacteria but not in fungi, and it is possible that endophytic fungi are more relevant to bioactive metabolites in medicinal plants (Jalgaonwala et al., 2011; Jia et al., 2016). In addition, compartment significantly affected the community structure of *P.n.*-associated microbiomes, which is consistent with previous results obtained in medicinal plants such as *Panax ginseng*, *Macleaya cordata,* and *Pseudowintera colorata* (Chowdhury et al., 2017; Purushotham et al., 2020; Lei et al., 2021) and may stem from the fact that the synthesis and transformation of secondary metabolites in different organs of medicinal plants are closely related to endophytes (Zhao et al., 2016; Song et al., 2017a).

Pine species, as a factor second to compartment had no significant effect on the diversity of microbiomes (bacteria and fungi) of *P.n.*, but changed the community structure of *P.n.* fungi. The differences in community richness (Chaos index) of *P.n.*-associated microbiomes in different pine species systems indicated that pine species changed rare populations rather than abundant microbial species (Shannon, 1948; Chao and Yang, 1993). Previous studies showed that tree rows in agroforestry systems significantly affected fungi rather than bacteria (Beule and Karlovsky, 2021), which is consistent with this study. This may be because soil fungi are more sensitive to changes in plant litter than bacteria (Yang et al., 2017), and bacteria are more resistant to disturbance than fungi in terms of structure, diversity, and biomass (Uroz et al., 2016). Additionally, endophytic bacteria are more easily influenced by the host plants than the soil source, and therefore plant-associated bacteria are more stable (Bonito et al., 2014).

*P.n.* genotype did not significantly affect the diversity and community structure of *P.n.*-associated microbiomes, which is consistent with the results obtained from previous studies on the assembly of quinoa-associated microbiomes (Cai et al., 2020). Some studies showed that the effect of host genotype on microbial community structure was more pronounced when plants have distant phylogenetic affiliations (Brassicaceae and Poaceae, for example) (Glynou et al., 2018), whereas the two *P.n.* genotypes in this study differ slightly, so the effect of genotype was limited.

### Pine and *P.n.*-associated microbial assembly driven by different factors

Microbial assembly associated with *P.n.* and pine were influenced by different factors. Correlation analysis and RDA analysis showed that the main influencing factor for both pine and *P.n.* bacterial assembly was total potassium in the soil, while those for fungal community assembly of pine and *P.n.* were plant N and soil water content. Water content affects the community structure of fungi and has been verified in many studies (Kaisermann et al., 2015; Supramaniam et al., 2016), and some microbiomes involved in the potassium cycle related to potassium may play an important role in plant potassium uptake (Meena et al., 2014). Additionally, plant microbial community composition was significantly corrlated not only with soil N content (Harrison et al., 2007; Lagomarsino et al., 2007; Farrer and Suding, 2016), but also with plant N, because fungi can assist plants in accessing soil N (Adesemoye et al., 2008; Hardoim et al., 2015). However, bacterial community assembly of pine was also affected by ammonium nitrogen and soil water content, while *P.n.* bacterial assembly was influenced by nitrate nitrogen and plant water content. Previous studies showed that variations in soil water content were more likely to affect the bacterial community associated with oaks than with grasses (Fierer et al., 2003), which is consistent with this study. This is probably because bacteria associated with woody plants are less exposed to drought stress (Fierer et al., 2003), while *P.n.* as an understory herb itself is less susceptible to water loss (Chen and Cao, 2014), and the associated bacteria are more sensitive to the water content. The nitrogen preferences of pine- and *P.n.*-associated bacteria may stem from the different choices of woody and herbaceous plants in decomposing and utilizing nitrogen sources. Pine, as a coniferous species, has a large accumulation of lignin and secondary metabolites in the understory layer, which limits nitrogen nitrification (Peng et al., 2006), whereas herbaceous plants prefer to absorb nitrate nitrogen, a directly available nitrogen source (Bedell et al., 1999).

### Composition of microbiomes associated with pine trees and *P.n.* under agroforestry systems

Land conversion did not change the community composition of pine- and *P.n.*-associated microbiomes. The pine-associated bacteria were mainly composed of Proteobacteria, Actinobacteria, Acidobacteria, and Chloroflexi, while the pine-associated fungi were mainly composed of Basidiomycota and Ascomycota, which was consistent with the results obtained by previous studies on pine-dominated forest soil microbiomes (Jiang et al., 2021; Li et al., 2021). The different compartments of the pine trees presented a greater proportion of the Proteobacteria in the roots and Actinobacteria in the soil. Probably because Proteobacteria, as a fast-growing eutrophic group in bacteria, can survive sufficient instability and rapidly propagate in substrates, its relative dominance is particularly pronounced in the root, an important organ of plant nutrient uptake (Lundberg et al., 2012; Edwards et al., 2015; Zhang et al., 2016). In contrast, actinomycetes were able to assist in the decomposition of the more massive plant litter through hyphae, and soil is more conducive to their reproduction than the internal plant environment (Dang et al., 2017). Additionally, the Ascomycota abundance of the pine fungi increased, while the Basidiomycota abundance decreased. This may be because of an increase in the herbaceous component of the plant litter and a relative decrease in the low-quality litter components with high lignification during land conversion (Purahong et al., 2016).

In addition, bacteria associated with *P.n.* included Proteobacteria, Actinobacteria, and Acidobacteria, while fungi mainly included Basidiomycota and Ascomycota. Ascomycota was dominant in the endosphere of *P.n.*, and the results were consistent with previous studies in which the most abundant genera of endophytic fungi of 29 medicinal herbs were all Ascomycota. This may be because the synthesis of antioxidant and antimicrobial active metabolites in medicinal herbs is associated with some species of Ascomycota (Huang et al., 2008).

### Possible transmission of endophytes between pine and *P.n.* in *P.n.*-pine agroforestry systems

There were new taxa of endophyte species added to pine trees in the *P.k./P.a.* -*P.n.* agroforestry systems. The increased species of endophytes of *P.k.* and *P.a.* share common populations with endophytes of *P.n.* (bacteria: 73.9, 93.3%, fungi: 68.75, 93.9%). The results showed that the endophytes (roots, stems, and leaves) of *P.n.* spread to the pine roots, which is consistent with the *Calliandra calothyrsus*-*Phaseolus vulgaris* agroforestry system and *Spartina alterniflora*-*Kandelia obovata* agroforestry system (Ingleby et al., 2007; Hong et al., 2015). This is because interplant interactions are critical for shaping the plant flora (Hong et al., 2015). Additionally, the existence of complex plant-microbial-plant networks within agroforestry systems formed by interplanting trees and crops can significantly reshape the composition of endophytes (Mei et al., 2022). We speculated that most of the transferred taxa are opportunistic endophytes, which have a certain probability of vertical transmission by factors such as plant internal factors (material transport, metabolism) and environmental factors (rain, wind) or horizontal diffusion (Tadych et al., 2007; Hardoim et al., 2015).

In addition, the endophyte that *P.n.* transferred to pine trees include multiple species of beneficial microbiomes. Some bacterial strains of Massilia and Marmoricola have the capacity to produce IAA, provide siderophores and antagonize pathogenic microbes *in vitro* (Ofek et al., 2012; Jiang et al., 2017), and strains of Herbaspirillum, Tardiphaga and Telmatospirllum are involved in biological nitrogen fixation (Monteiro et al., 2012) and slow-growing nitrogen fixation and participate in the sulfur cycle (Perley and Stowe, 1966; Hausmann et al., 2018), respectively. The increased beneficial fungi included Umbelopsis, Xylara, Geminibasidium, Inocybe, Alatospora, and Pleotricholadium, with the functions of participating in the transformation of lipids and polysaccharides (Dourou et al., 2017), synthesizing antioxidant active compounds (Liu et al., 2007), participating in the carbon and nitrogen cycle (Pulido-Chavez et al., 2021) and decomposing and utilizing functions of organic matter (Feckler et al., 2017; Wang et al., 2021). In summary, these beneficial microbiomes were delivered by *P.n.* to pine trees were favorable to the growth of pine trees. Therefore, the agroforestry organic economic cultivation of *P.n.*-pine trees is sustainable.

## Conclusion

In conclusion, the conversion of the two pure pine forests (*P.k.* and *P.a.)* to the *P.n.*-pine agroforestry systems significantly changed the diversity, but not the community structure, of pine-associated fungi. The community structure, but not the diversity, of *P.n.*-associated fungi was significantly changed. Fungi were more sensitive to alterations in both plant-associated factors than bacteria. Main effect analysis showed that compartment but not genotype was the driving factor affecting *Panax notoginseng* and pine, but *Panax notoginseng* cultivation also significantly influenced the assembly of pine related microbiomes. In addition, a diffuse spread of *P.n.* endophytes into the pine roots, and beneficial microbiomes (Massilia, Marmoricola, Herbaspirillum, etc.) increased in pine roots. Therefore, the different assembly mechanisms of *Panax notoginseng* and pine microbiomes functioned as an important role in the *Panax notoginseng*-pine agroforestry systems and were the basis for the sustainable development of the *Panax notoginseng*-pine agroforestry systems.

## Data availability statement

The datasets presented in this study can be found in online repositories. The names of the repository/repositories and accession number(s) can be found at: https://www.ncbi.nlm.nih.gov/, PRJNA821648 and PRJNA821834.

## Author contributions

WJ: methodology, formal analysis, investigation, data curation, writing - original draft, writing - review and editing, and visualization. SW: methodology, formal analysis, conceptualization, investigation, writing - original draft, and writing - review and editing. XH: project administration, funding acquisition, conceptualization, and writing - review and editing. XZ: methodology, investigation, and writing -review and editing. All authors contributed to the article and approved the submitted version.

## Funding

This work was supported by the China Agriculture Research System (CARS-21), the Major Science and Technology Project of Yunnan Province (202102AE090042, 2019ZG0901, 2021Y250) and the Kunming Science and Technology Bureau (2021JH002).

## Conflict of interest

The authors declare that the research was conducted in the absence of any commercial or financial relationships that could be construed as a potential conflict of interest.

## Publisher’s note

All claims expressed in this article are solely those of the authors and do not necessarily represent those of their affiliated organizations, or those of the publisher, the editors and the reviewers. Any product that may be evaluated in this article, or claim that may be made by its manufacturer, is not guaranteed or endorsed by the publisher.

## Supplementary material

SUPPLEMENTARY TABLE S1 Vegetation type of two different pine forests.

SUPPLEMENTARY TABLE S2 PerMANOVA (Permutational multivariate analysis) indicated the relative contributions of genotype (G), planting *P.n.* or not (Pn) and their interactions on bacterial and fungal alpha- diversity among different compartments (bulk and rhizosphere soils, and root of pine trees). Significant effects (*p*<0.05) are shown in bold. *p* < 0.05, ***p* < 0.01, ****p* < 0.001.

SUPPLEMENTARY TABLE S3 Pearson correlations (r value) between bacterial and fungal ɑ diversity of pine-associated microbes (Shannon, Chao 1, Shannoneven) and plant and soil variables among different compartments (bulk, rhizosphere soil and roots of pine trees). Significant effects (*p*<0.05) are shown in bold. Abbreviations: F-sha, shannon index of fungi; F-Chao1, Chao1 of fungi; F-even, shannoneven index of fungi; B-sha, shannon index of bacteria; B-Chao1, Chao1 of bacteria; B-even, shannoneven index of bacteria; STK, soil total potassium; STN, soil total nitrogen; STP, soil phosphorous; NH_4_^+^-N, soil ammonium nitrogen; NO_3_^-^-N, soil nitrate nitrogen; SWC, soil water content; EC, soil conductivity.

SUPPLEMENTARY TABLE S4 PerMANOVA (Permutational multivariate analysis) indicated the relative contributions of genotype (G), pine tree species (Ps) and their interactions on bacterial and fungal alpha diversity among different compartments (rhizosphere soils, roots, stems, leaves of *P.n.*). *p* < 0.05, ***p* < 0.01, ****p* < 0.001.

SUPPLEMENTARY TABLE S5 Pearson correlations (r value) between bacterial and fungal α-diversity of *P.n.*-associated microbes (Shannon, Chao 1, Shannoneven) and plant and soil variables. Significant effects (*p* < 0.05) are shown in bold. Abbreviations of soil variables are as defined in Supplementary Table S3. Abbreviations: F-sha, shannon index of fungi; F-Chao1, Chao1 of fungi; F-even, shannoneven index of fungi; B-sha, shannon index of bacteria; B-Chao1, Chao1 of bacteria; B-even, shannoneven index of bacteria; PWC, plant water content; P_Fw, plant fresh weight; P_Dw, plant dry weight; PTN, plant total nitrogen; PTP, plant total phosphorous.

SUPPLEMENTARY TABLE S6 PerMANOVA based on WUF revealing the relative contributions of genotype (G), compartment (C) and planting *P.n.* or not (Pn) on bacterial and fungal variations across all samples and in each compartment of pine-associated microbes. Significant levels: *p* < 0.05, ***p* < 0.01, ****p* < 0.001.

SUPPLEMENTARY TABLE S7 PerMANOVA based on WUF revealing the relative contributions of genotype (G), compartment (C) and pine tree species (Ps) on bacterial and fungal variations across all samples and in each P.n.-asscioated compartment (rhizosphere, root, stem, leaves). Significant levels: **p* < 0.05, ***p* < 0.01, ****p* < 0.001.

SUPPLEMENTARY FIGURE S1 PCoA plots based on WUF (weighted unifrac distance) of bacterial and fungal OTU showing the variation in each compartment of pine-associated microbes (root, rhizosphere, between).

SUPPLEMENTARY FIGURE S2 PCoA plots based on WUF of bacterial and fungal OTU showing the variation in each compartment of *P.n.*-associated microbes (rhizosphere, root, stem, leaves).

SUPPLEMENTARY FIGURE S3 Venn Diagram exhibiting the overlap among the *P.n.* endophyte and the pine root endophyte of *P.n.*-pine agroforestry systems and the root endophyte of pure pine forests.

SUPPLEMENTARY FIGURE S4 Student *t*-test bar plots for different compartments of bacterial community of *P.k.* on genus level. PP, pure pine forest, GP, agroforestry system.

SUPPLEMENTARY FIGURE S5 Student *t*-test bar plots for different compartments of bacterial community of *P.a.* on genus level. PP, pure pine forest, GP, agroforestry system.

SUPPLEMENTARY FIGURE S6 Student *t*-test bar plots for different compartments of fungal community of *P.k.* on genus level. PP, pure pine forest, GP, agroforestry system.

SUPPLEMENTARY FIGURE S7 Student *t*-test bar plots for different compartments of fungal community of *P.a.* on genus level. PP, pure pine forest, GP, agroforestry system.

SUPPLEMENTARY FIGURE S8 Student *t*-test bar plots for different compartments of fungal community of *P.n.* on genus level. Pk, *P.n.*-*P.k.* agroforestry system, Pa, *P.n.*-*P.a.* agroforestry system.

SUPPLEMENTARY FIGURE S9 Student *t*-test bar plots for different compartments of fungal community of *P.n.* on genus level. Pk, *P.n.*-*P.k.* agroforestry system, Pa, *P.n.*-*P.a.* agroforestry system.
