# Supplementary material for: Different factors drive the assembly of pine and Panax notoginseng-associated microbiomes in Panax notoginseng-pine agroforestry systems
Source: Front Microbiol. 2022 Nov 14;13:1018989. doi: 10.3389/fmicb.2022.1018989 (PMC9702986; doi:10.3389/fmicb.2022.1018989)
Supplement: SUPPLEMENTARY TABLE S1 — Vegetation type of two different pine forests. [file Data_Sheet_1.pdf]

Supplementary Material

1. Supplementary methods: PCR amplification and DNA sequencing

DNA was extracted from soil samples (0.5 g each) using the No, 12888.100 Qiagen DNeasy PowerSoil Kit (MP Biomedicals, Solon, CA, USA) according to the manufacturer's instructions and from 0.5 g of plant tissue (pine roots and *P.n.* roots, stems and leaves) using the Qiangen DNeasy Plant Kit (MP Biomedicals, Solon, CA, USA). DNA purity and concentration were estimated by NanoDrop2000 (Thermo Fisher Scientific, Waltham, MA, USA). The quality and quantity of DNA was checked on 1% agarose gels to verify extraction success for subsequent analysis.

The V5-V6 region of the 16S rRNA was amplified with 799F (Chelius and Triplett, 2001) - 1193R (Bodenhausen et al, 2013), and the conditions were as follows: an initial denaturation at 95 °C for 2 min; followed by 35 cycles of 95 °C for 30 s, 30 s at 55 °C, 72 °Cfor 45 s, and a final extraction at 72 °C for 10 min. The ITS region of the fungi was amplified with ITS1 (Gardes and Bruns, 1993) - ITS2 (White et al, 1990), and the conditions were as follows: an initial denaturation at 95 °C for 2 min; followed by 35 cycles of 95 °Cfor 30 s, 30 s at 53 °C, 72 °C for 45 s, and a final extraction at 72 °C for 10 min. The primer sequences are listed below.

Name	Sequences	Reference
799F<br>1193R<br>ITS1F<br>ITS2R	AACMGGATTAGATACCCKG<br>ACGTCATCCCCACCTTCC<br>CTTGGTCATTTAGAGGAAGTAA<br>GCTGCGTTCTTCATCGATGC	Chelius et al, 2001; Bodenhausen et al, 2013; Gardes and Bruns, 1993; White et al, 1990

A 20 μl reaction system was used for PCR, containing 10 ng of template DNA (Template DNA), 4 μL of 5 x FastPfu Buffer, 2 μL of 2.5 mM dNTPs, 0.8 μL of Forward Primer (5 μM) and Reverse Primer (5 μM), 0.4 μL of FastPfu Polymerase, 0.2 μL of BSA, and the rest of the volume was made up with the obtained ddH_2O. Our bacterial 16S rRNA and fungal ITS genes sequencing was performed on the Illumina MiSeq PE300 platform. After PCR product identification and purification, Miseq library construction was performed using the NEXTFLEX® Rapid DNA-Seq Kit (Bioo Scientific, Austin, TX, USA) followed by quality control of the raw sequenced sequences using Trimmomatic 0.32 (Bolger et al., 2014) and splicing using FLASH 1.2.11 (Magoč and Salzberg, 2011) and UPARSE 7.1 (Edgar, 2013),,OTU clustering was performed using the usearch algorithm based on 97% similarity, and chimeras were removed using UCHIME 4.1 (Edgar et al, 2011) to obtain OTU tables. Finally,

each sequence was annotated for species classification using the RDP classifier (http://rdp.cme.msu.edu/, Wang et al., 2007), with bacteria compared to the Silva database (SSU128, McDonald et al., 2012) and fungi compared to the Unite (Release 8.0 http://unite.ut.ee/index.php) database (Abarenkov et al, 2010). A comparison threshold of 70% was set. OTUs assigned to chloroplasts and mitochondria were removed prior to subsequent analysis. We obtained high quality reads of 1921312 and 6064848 from 16SrRNA and ITS sequences with an average trim length of 243bp and 376bp, respectively. We normalized the sequences prior to subsequent analysis in order to eliminate differences in sequencing depth with the "vegan" package of R4.1.0 (Dixon, 2003).

Reference

Abarenkov, K., Nilsson, R. H., Larsson, K. H., Alexander, I. J., Eberhardt, U., Erland, S., Høiland, K.,Kjøller, R., Larsson, E., Pennanen, T., Sen, R., F. S.Taylor, A., Tedersoo, L., Ursing, M. B.,Vrålstad, T., Liimatainen, K., Peintner, U., Kõljalg, U., 2010. The UNITE database for molecular identification of fungi–recent updates and future perspectives. The New Phytologist, 186(2), 281-285.

Bodenhausen, N., Horton, M. W., Bergelson, J., 2013. Bacterial communities associated with the leaves and the roots of Arabidopsis thaliana. PloS one, 8(2), e56329. https://doi.org/10.1371/journal.pone.0056329

Bolger, A. M., Lohse, M., Usadel, B., 2014. Trimmomatic: a flexible trimmer for Illumina sequence data. Bioinformatics, 30(15), 2114-2120. https://doi.org/10.1093/bioinformatics/btu170

Chelius, M. K., Triplett, E. W., 2001. The Diversity of Archaea and Bacteria in Association with the Roots of *Zea mays* L. Microbial ecology, 252-263.

Dixon P., 2003. VEGAN, a package of R functions for community ecology. Journal of Vegetation Science, 14(6): 927-930. https://doi.org/10.1111/j.1654-1103.2003.tb02228.x

Duran, P., Thiergart, T., Garrido-Oter, R., Agler, M., & Hacquard, S., 2018. Microbial interkingdom interactions in roots promote arabidopsis survival. Cell, 175(4), 973-983.e14.

Edgar, R. C., 2013. UPARSE: highly accurate OTU sequences from microbial amplicon reads. Nature methods, 10(10), 996-998. DOI: 10.1038/nmeth.2604

Edgar, R. C., Haas, B. J., Clemente, J. C., Quince, C., Knight, R., 2011. UCHIME improves sensitivity and speed of chimera detection. Bioinformatics, 27(16),

2194-2200. https://doi.org/10.1093/bioinformatics/btr381

Gardes, M., Bruns, T. D., 1993. ITS primers with enhanced specificity for basidiomycetes-application to the identification of mycorrhizae and rusts. Molecular ecology, 2(2), 113-118. https://doi.org/10.1111/j.1365-294X.1993.tb00005.x

Magoč, T., Salzberg, S. L., 2011. FLASH: fast length adjustment of short reads to improve genome assemblies. Bioinformatics, 27(21), 2957-2963. https://doi.org/10.1093/bioinformatics/btr507

McDonald, D., Price, M. N., Goodrich, J., Nawrocki, E. P., DeSantis, T. Z., Probst, A., Andersen, G. L., Knight, R., Hugenholtz, P., 2012. An improved Greengenes taxonomy with explicit ranks for ecological and evolutionary analyses of bacteria and archaea. The ISME journal, 6(3), 610-618. DOI: 10.1038/ismej.2011.139

Wang, Q., Garrity, G.M., Tiedje, J.M., Cole, J.R., 2007. Naive Bayesian classifier for rapid assignment of rRNA sequences into the new bacterial taxonomy. Appl Environm Microb 73:5261–5267. DOI: https://doi.org/10.1128/AEM.00062-07

White, T. J., Bruns, T., Lee, S. J. W. T., Taylor, J., 1990. Amplification and direct sequencing of fungal ribosomal RNA genes for phylogenetics. PCR protocols: a guide to methods and applications, 18(1), 315-322.

Supplementary Table 1. Vegetation type of two different pine forests.

Vegetation Types	*Pinus kesiya* forests	*Pinus armandii* forests
Arbor	*Pinus kesiya*	*Pinus armandii*
Shrub	*Breynia fruticosa*	*Berberis atrocarpa*
Shrub	*Ternstroemia simaoensis*	*Euonymus alatus*
Liana	/	*Schisandra chinensis*
Herb	*Ageratina adenophora*	*Ageratina adenophora*
	Imperata cylindrica	*Rubus parvifolius*
	Hedyotis uncinella	*Anisocampium cuspidatum*
	Pteris ensiformis	
	Sophora flavescens	

Supplementary Table 2. PerMANOVA (Permutational multivariate analysis) indicated the relative contributions of genotype (G), planting *P.n.* or not (Pn) and their interactions on bacterial and fungal alpha- diversity among different compartments (bulk and rhizosphere soils, and root of pine trees). Significant effects ($p < 0.05$) are shown in bold. * $p < 0.05$, ** $p < 0.01$, ***, $p < 0.001$

			Root		Rhizosphere		Between	
	Sources	df	F	Sig.	F	Sig	F	Sig.
Bacteria								
Shannon	G	1	0.694	0.429	0.109	0.750	1.249	0.295
	Pn	1	0.09	0.115	2.229	0.521	1.318	0.298
	G×Pn	1	0.578	0.469	10.139	**0.013****	35.287	**0.000*****
Model r2			0.149		0.909		0.585	
Chao1	G	1	3.214	0.111	2.674	0.142	3.827	0.088
	Pn	1	3.335	0.109	3.217	0.111	1.615	0.24
	G×Pn	1	2.132	0.182	2.842	0.130	2.135	0.182
Model r2			0.624		0.611		0.343	
Shannon even	G	1	0.567	0.473	1.409	0.284	5.107	0.061
	Pn	1	0.408	0.541	2.389	0.164	3.468	0.116
	G×Pn	1	0.923	0.365	31.162	**0.001*****	54.13	**0.000*****
Model r2			0.192		0.915		0.837	
Fungi								
Shannon	G	1	1.254	0.295	33.087	**0.000*****	85.153	**0.000*****
	Pn	1	0.184	0.679	231.8	**0.000**	1.753	0.222

					17	***		
	G×Pn	1	0.100	0.760	0.136	0.722	5.547	0.060
Model r2			0.161		0.920		0.960	
Chao1	G	1	0.525	0.490	5.325	**0.042***	4.987	**0.043***
	Pn	1	4.177	0.057	26.279	**0.001*****	4.864	0.055
	G×Pn	1	3.478	0.099	0.094	0.767	3.254	0.105
Model r2			0.550		0.715		0.736	
Shannon even	G	1	1.491	0.257	5.065	0.055	28.162	**0.002****
	Pn	1	0.01	0.922	50.772	**0.000*****	0.195	0.671
	G×Pn	1	0.368	0.561	0.092	0.769	0.409	0.540
Model r2			0.189		0.879		0.828	

Supplementary Table 3. Pearson correlations (r value) between bacterial and fungal α diversity of pine-associated microbes (Shannon, Chao 1, Shannoneven) and plant and soil variables among different compartments (bulk, rhizosphere soil and roots of pine trees). Significant effects ($p < 0.05$) are shown in bold. * $p < 0.05$, ** $p < 0.01$

	F-Sha	F-Chao1	F-even	B-Sha	B-Chao	B-even
Rhizosphere						
STK	**0.587***	0.439	**0.604***	0.426	0.529	0.195
STN	**0.720****	**0.700***	**0.630***	**0.677***	0.303	**0.924****
STP	-0.426	-0.283	-0.462	-0.307	-0.489	-0.017
NH4+-N	0.348	0.290	0.302	**0.796****	0.662*	**0.727****
NO3--N	0.079	-0.041	0.144	0.076	0.380	-0.294
SWC	**0.596***	0.508	**0.615***	**0.524***	**0.549***	**0.584***
pH	**0.824****	**0.738****	**0.809****	0.083	-0.021	0.191
EC	-0.434	-0.383	-0.388	-0.379	-0.294	-0.382
Root						
STK	0.296	0.442	0.209	-0.021	**-0.757****	0.081
STN	-0.317	-0.392	-0.238	0.046	**0.765****	-0.054
STP	0.045	-0.132	0.062	-0.047	-0.056	-0.046
NH4+-N	0.253	-0.156	0.287	-0.234	-0.128	-0.230
NO3--N	0.329	0.240	0.277	-0.100	-0.688	-0.015
SWC	-0.044	0.717	-0.184	0.298	-0.567	0.395
pH	-0.076	**0.734****	-0.215	0.313	-0.499	0.403
EC	-0.086	-0.673	0.042	-0.187	**0.645***	-0.288
RN	**0.847****	**0.897****	**0.681****	**0.915****	**0.943****	**0.863****
Bulk soil						
STK	-0.389	-0.173	-0.422	-0.473	-0.073	**-0.650***
STN	**0.734****	0.523	**0.699***	0.039	-0.250	0.217
STP	**0.895****	**0.697***	**0.806****	-0.470	-0.551	-0.336
NH_4^+-N	0.372	-0.451	0.502	-0.305	-0.415	-0.110
NO_3^--N	**-0.956****	-0.450	**-0.930****	0.509	**0.645***	0.306
SWC	0.175	**0.755****	0.024	0.092	0.093	0.014
pH	**0.653***	0.301	**0.601***	**-0.947****	**-0.739****	**-0.890****
EC	0.275	0.457	0.152	**-0.793****	**-0.449**	**-0.892****
	-0.426	-0.283	-0.462	-0.307	-0.489	-0.017

Abbreviations: F-sha, shannon index of fungi; F-Chao1, Chao1 of fungi; F-even, shannoneven index of fungi; B-sha, shannon index of bacteria; B-Chao1, Chao1 of bacteria; B-even, shannoneven index of bacteria; STK, soil total potassium; STN, soil total nitrogen; STP, soil phosphorous; NH_4^+-N, soil ammonium nitrogen; NO_3^--N, soil nitrate nitrogen; SWC, soil water content; EC, soil conductivity.

Supplementary Table 4. PerMANOVA (Permutational multivariate analysis) indicated the relative contributions of genotype (G), pine tree species (Ps) and their interactions on bacterial and fungal alpha diversity among different compartments (rhizosphere soils, roots, stems, leaves of *P.n.*). * $p < 0.05$, ** $p < 0.01$, ***, $p < 0.001$

			Rhizosphere		Root		Stem		Leaves	
	Sources	df	F	Sig.	F	Sig	F	Sig.	F	Sig
Bacteria										
Shannon	G	1	1.385	0.273	0.985	0.350	0.663	0.808	0.225	0.648
	Ps	1	0.054	0.822	1.894	0.206	3.660	0.092	0.280	0.872
	G×Ps	1	1.471	0.260	0.019	0.894	0.025	0.877	0.432	0.529
Model r2			0.267		0.266		0.319		0.079	
Chao1	G	1	2.426	0.158	0.159	0.700	3.686	0.091	0.966	0.355
	Ps	1	9.050	**0.017** **	3.588	0.095	0.001	0.979	10.649	**0.011** **
	G×Ps	1	2.359	0.163	0.750	0.412	0.464	0.515	1.609	0.240
Model r2			0.623		0.360		0.342		0.634	
Shannon even	G	1	1.471	0.260	1.471	0.316	0.025	0.877	0.432	0.529
	Ps	1	15.131	**0.005** **	15.131	0.057	0.503	0.498	0.789	0.400
	G×Ps	1	12.632	**0.007** **	12.632	0.824	0.213	0.657	0.250	0.631
Model r2			0.776		0.434		0.136		0.240	
Fungi										
Shannon	G	1	2.575	0.147	0.204	0.664	0.039	0.848	0.116	0.743
	Ps	1	5.041	0.550	0.111	0.747	0.122	0.736	3.529	0.097
	G×Ps	1	2.958	0.124	0.001	0.976	0.036	0.855	0.113	0.745
Model r2			0.569		0.038		0.024		0.320	
Chao1	G	1	0.190	0.675	0.331	0.581	4.219	0.072	5.637	**0.045** *
	Ps	1	86.725	**0.00*** **	2.820	0.132	3.246	0.109	17.084	**0.003** **
	G×Ps	1	0.324	0.585	0.227	0.646	0.002	0.965	0.069	0.799

Model r2			0.916		0.297		0.485		0.740	
Shannon even	G	1	2.464	0.155	0.038	0.851	0.023	0.883	0.194	0.671
	Ps	1	1.434	0.265	0.056	0.819	0.039	0.848	0.996	0.347
	G×Ps	1	3.097	0.116	0.002	0.967	0.015	0.906	0.269	0.618
Model r2			0.467		0.012		0.064		0.154	

Supplementary Table 5. Pearson correlations (r value) between bacterial and fungal α-diversity of *P.n.*-associated microbes (Shannon, Chao 1, Shannoneven) and plant and soil variables. Significant effects (p<0.05) are shown in bold. Abbreviations of soil variables are as defined in Table S3. * $p < 0.05$, ** $p < 0.01$

	X-Sha	X-Chao	X-even	F-Sha	F-Chao1	F-even
PWC	-0.576	-0.574	-0.488	-0.524	0.063	**0.685***
P_Fw	**0.862****	**0.792****	**0.629***	**-0.652***	-0.288	**-0.645***
P_Dw	**0.731****	**0.765****	-0.507	**-0.648****	-0.277	**-0.655****
PTN	-0.432	-0.450	-0.233	0.239	0.520	-0.152
PTP	-0.342	-0.440	-0.169	0.127	-0.388	-0.213
STK	0.312	-0.162	-0.569	0.212	0.554	-0.111
STN	0.272	-0.294	0.511	0.287	**0.796***	-0.147
STP	-0.680	-0.245	**-0.751***	0.237	-0.562	0.702
NH4+-N	0.227	0.124	0.305	-0.003	0.137	-0.158
NO3--N	0.587	-0.036	**0.786***	0.289	**0.858****	-0.211
SWC	-0.179	-0.390	-0.020	0.141	0.238	0.030
pH	-0.626	-0.417	-0.516	0.225	-0.381	0.570
EC	0.380	-0.107	0.586	0.273	**-0.800***	-0.213

Abbreviations: F-sha, shannon index of fungi; F-Chao1, Chao1 of fungi; F-even, shannoneven index of fungi; B-sha, shannon index of bacteria; B-Chao1, Chao1 of bacteria; B-even, shannoneven index of bacteria; PWC, plant water content; P_Fw, plant fresh weight; P_Dw, plant dry weight; PTN, plant total nitrogen; PTP, plant total phosphorous.

Supplementary Table 6. PerMANOVA based on WUF revealing the relative contributions of genotype (G), compartment (C) and planting *P.n.* or not (Pn) on bacterial and fungal variations across all samples and in each compartment of pine-associated microbes. Significant levels: * $p < 0.05$, ** $p < 0.01$, *** $p < 0.001$

Bacteria				Fungi			
Factors	R2	Pr(> F)	Sig	Factors	R2	Pr(> F)	Sig
Whole data							
G	0.0319	0.310		G	0.2699	**0.001**	***
Pn	0.0357	0.272		Pn	0.0464	0.126	
C	0.6204	**0.001**	***	C	0.2434	**0.001**	***
Root							
G	0.1137	0.300		G	0.5494	**0.003**	**
Pn	0.1403	0.179		Pn	0.0878	0.400	
Rhizosphere							
G	0.3083	**0.004**	**	G	0.5247	**0.003**	**
Pn	0.1843	0.120		Pn	0.2658	**0.041**	*
Between							
G	0.3823	**0.003**	**	G	0.6848	**0.003**	**
Pn	0.2429	**0.049**	*	Pn	0.1258	0.205	

Supplementary Table 7. PerMANOVA based on WUF revealing the relative contributions of genotype (G), compartment (C) and pine tree species (Ps) on bacterial and fungal variations across all samples and in each *P.n.*-asscioated compartment (rhizosphere, root, stem, leaves). Significant levels: * $p < 0.05$, ** $p < 0.01$, *** $p < 0.001$

Bacteria				Fungi			
Factors	R2	Pr(> F)	Sig	Factors	R2	Pr(> F)	Sig
Whole data							
G	0.0008	0.853		G	0.0162	0.649	
Ps	0.0044	0.625		Ps	0.0673	**0.005**	**
C	0.9683	**0.001**	***	C	0.4582	**0.001**	***
Rhizosphere							
G	0.1618	0.166		G	0.2149	0.109	
Ps	0.5347	**0.003**	**	Ps	0.5268	**0.003**	**
Root							
G	0.0077	0.513		G	0.1041	0.338	
Ps	0.1251	0.194		Ps	0.2577	**0.003**	**
Stem							

G	0.1248	0.247	G	0.0486	0.988	
Ps	0.0685	0.456	Ps	0.1715	**0.010**	*
Leaves						
G	0.0591	0.814	G	0.0618	0.812	
Ps	0.0630	0.816	Ps	0.1790	**0.008**	*

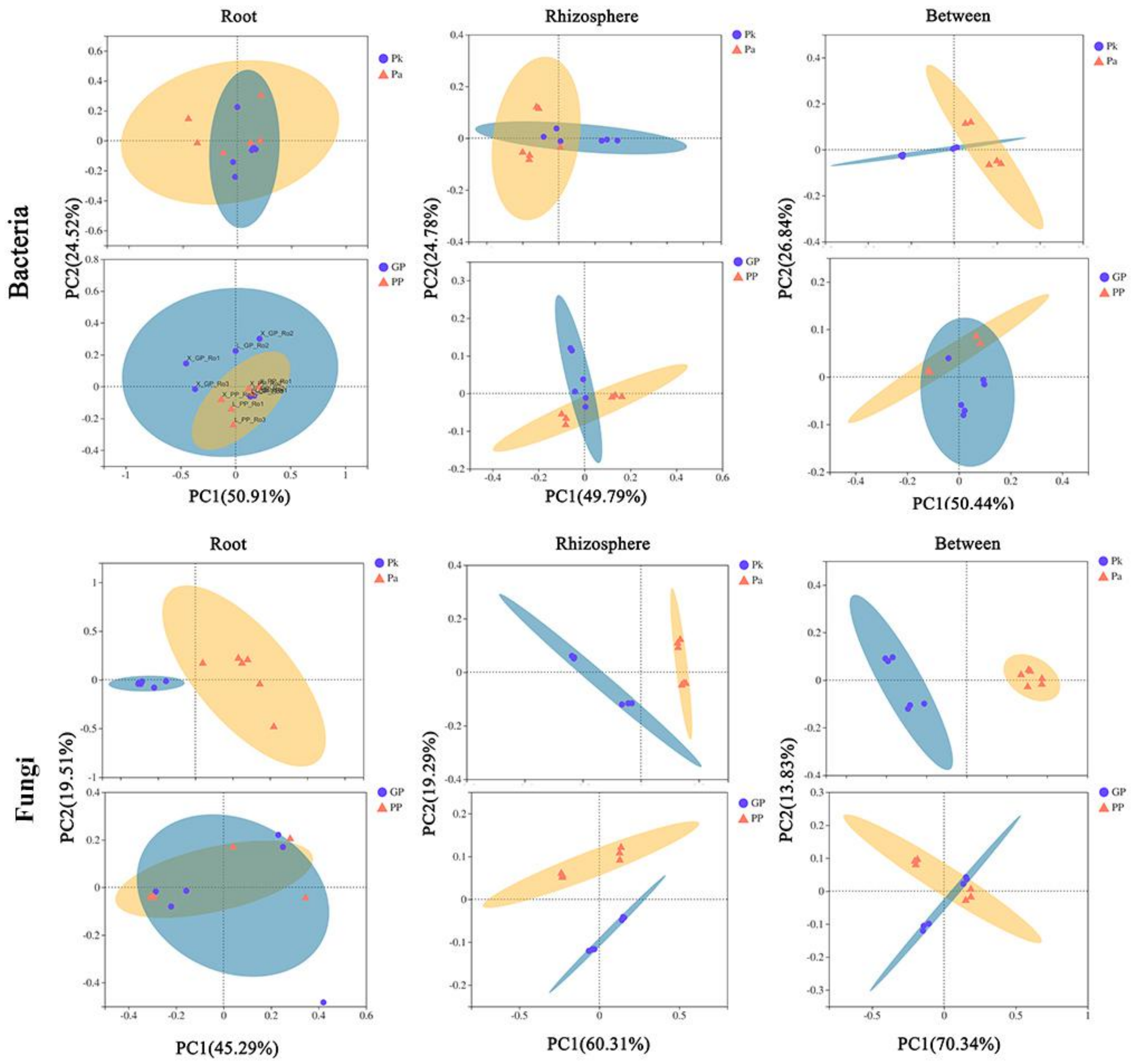

Supplementary Figure 1. PCoA plots based on WUF (weighted unifrac distance) of bacterial and fungal OTU showing the variation in each compartment of pine-associated microbes (root, rhizosphere, between).

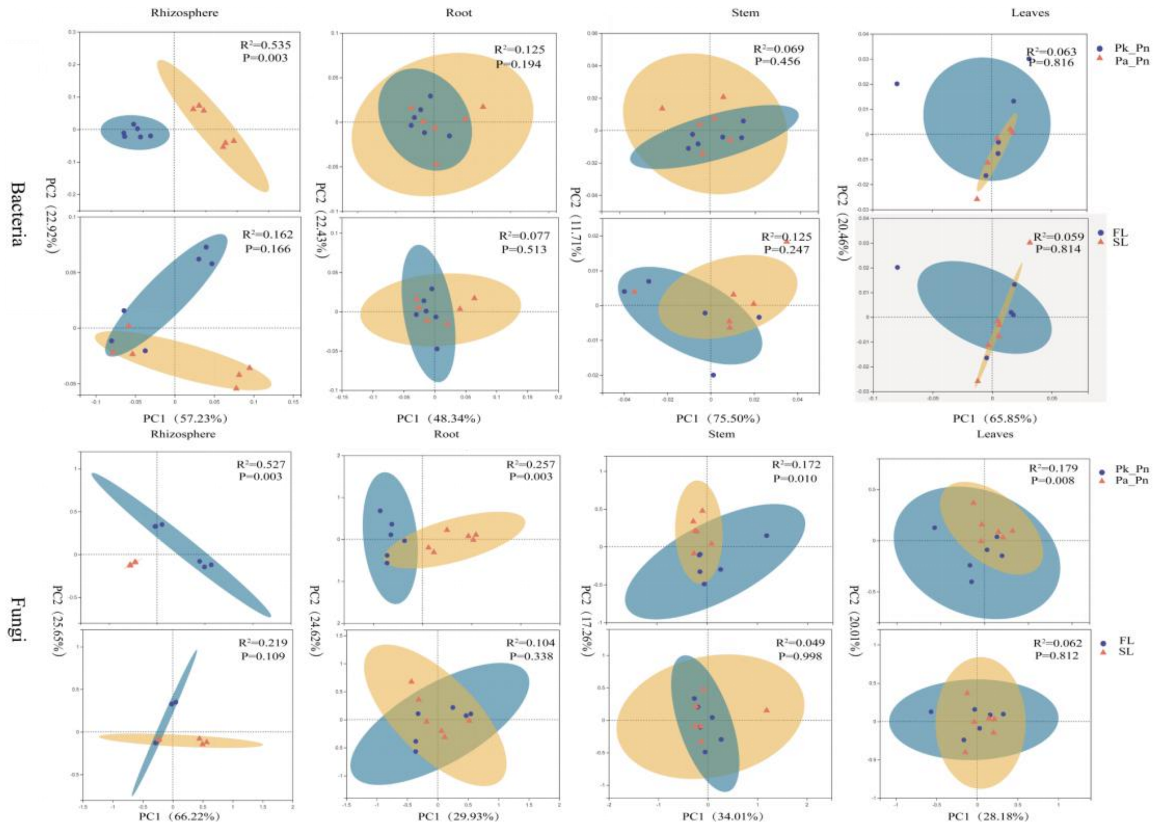

Supplementary Figure 2. PCoA plots based on WUF of bacterial and fungal OTU showing the variation in each compartment of *P.n.*-associated microbes (rhizosphere, root, stem, leaves).

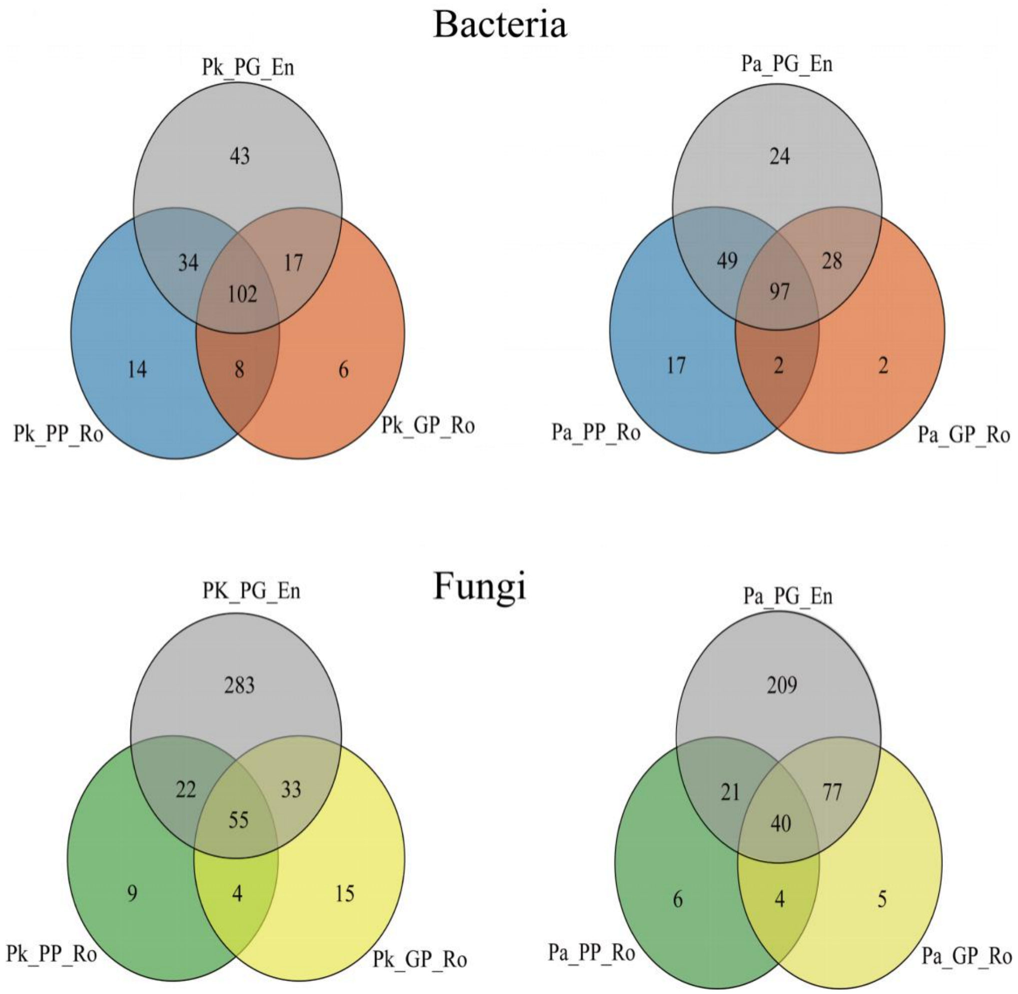

Supplementary Figure 3. Venn Diagram exhibiting the overlap among the *P.n.* endophyte and the pine root endophyte of *P.n.*-pine agroforestry systems and the root endophyte of pure pine forests.

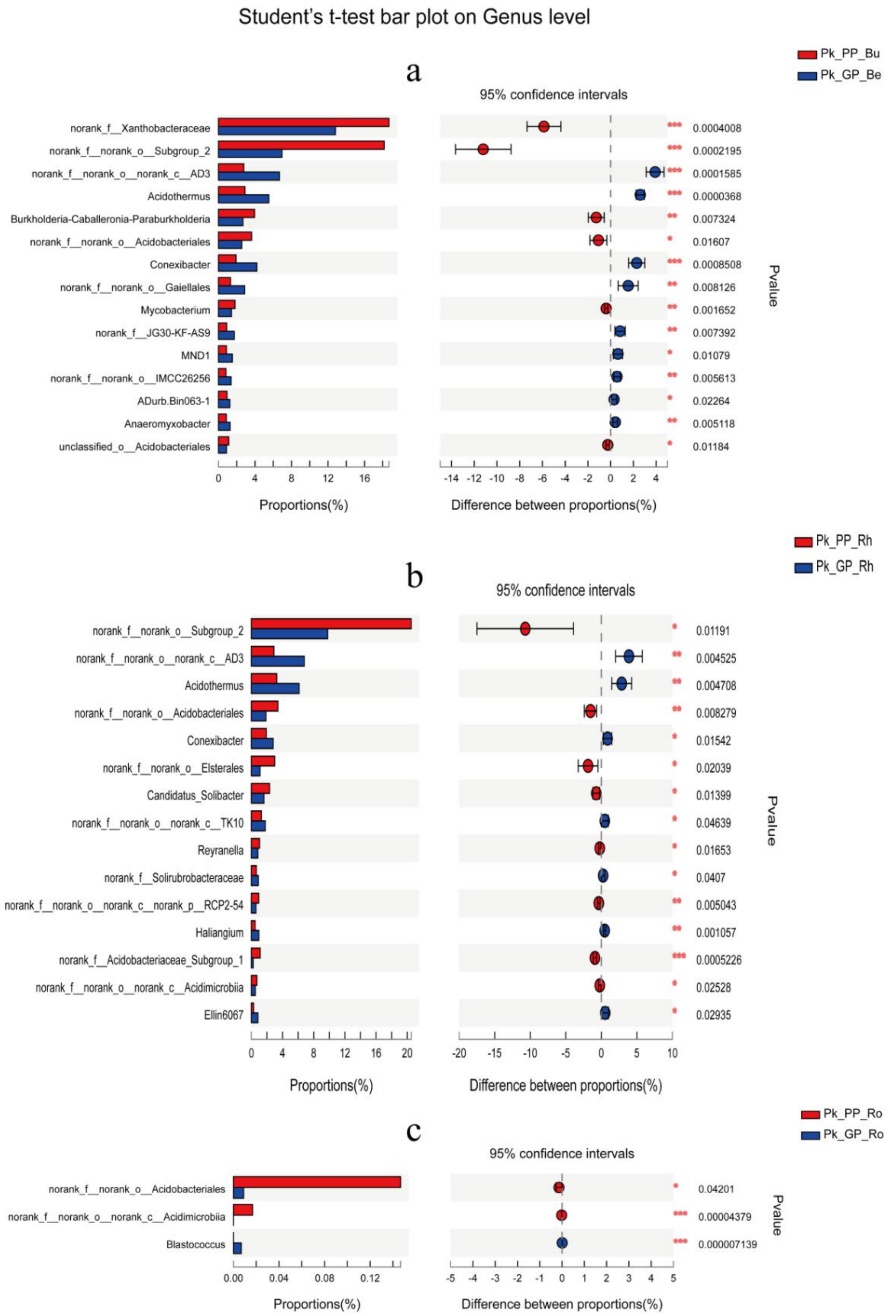

Supplementary Figure 4. Student t-test bar plots for different compartments of bacterial community of *P.k.* on genus level. PP, pure pine forest, GP, agroforestry system.

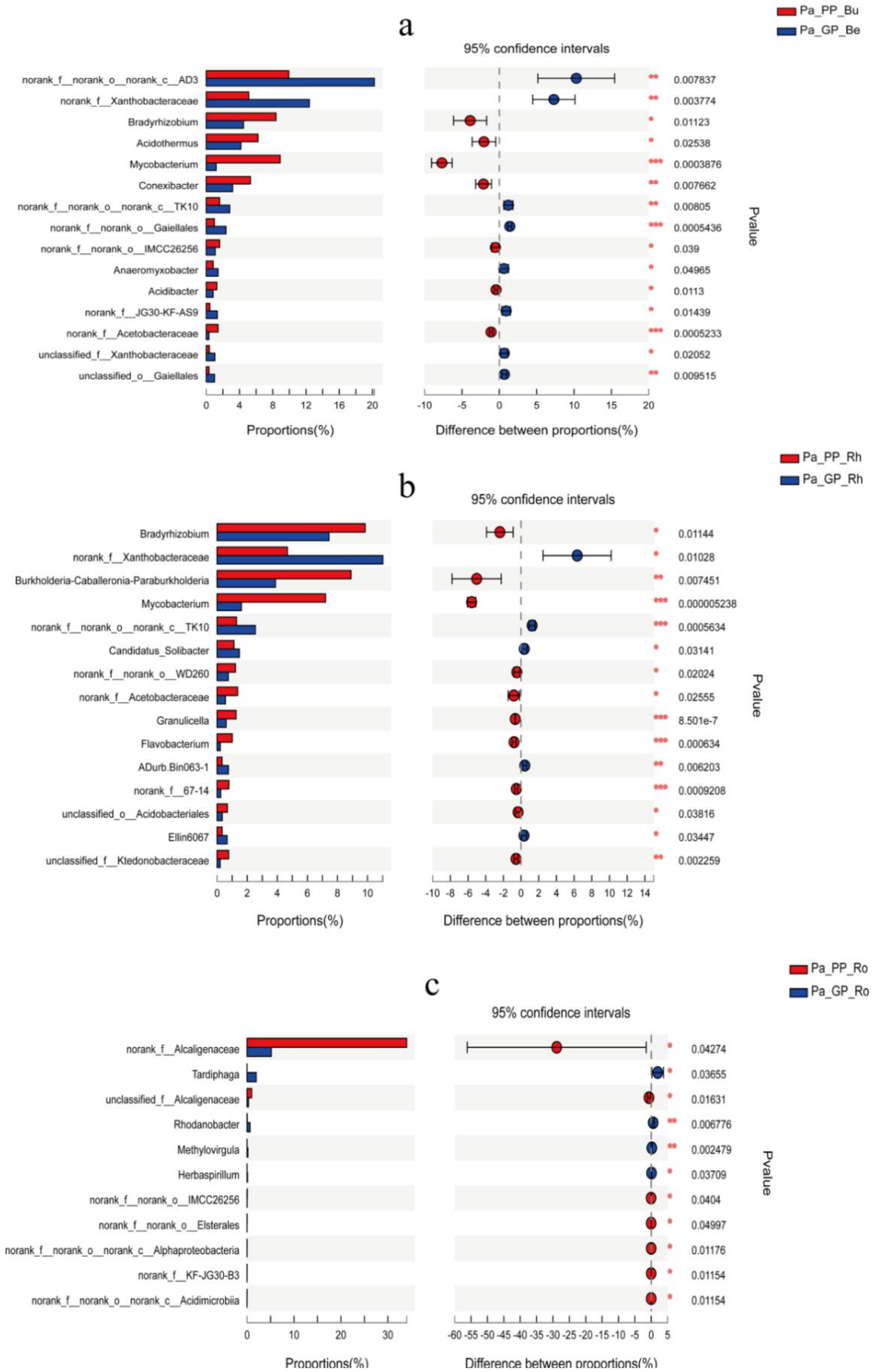

Supplementary Figure 5. Student t-test bar plots for different compartments of bacterial community of *P.a.* on genus level. PP, pure pine forest, GP, agroforestry system.

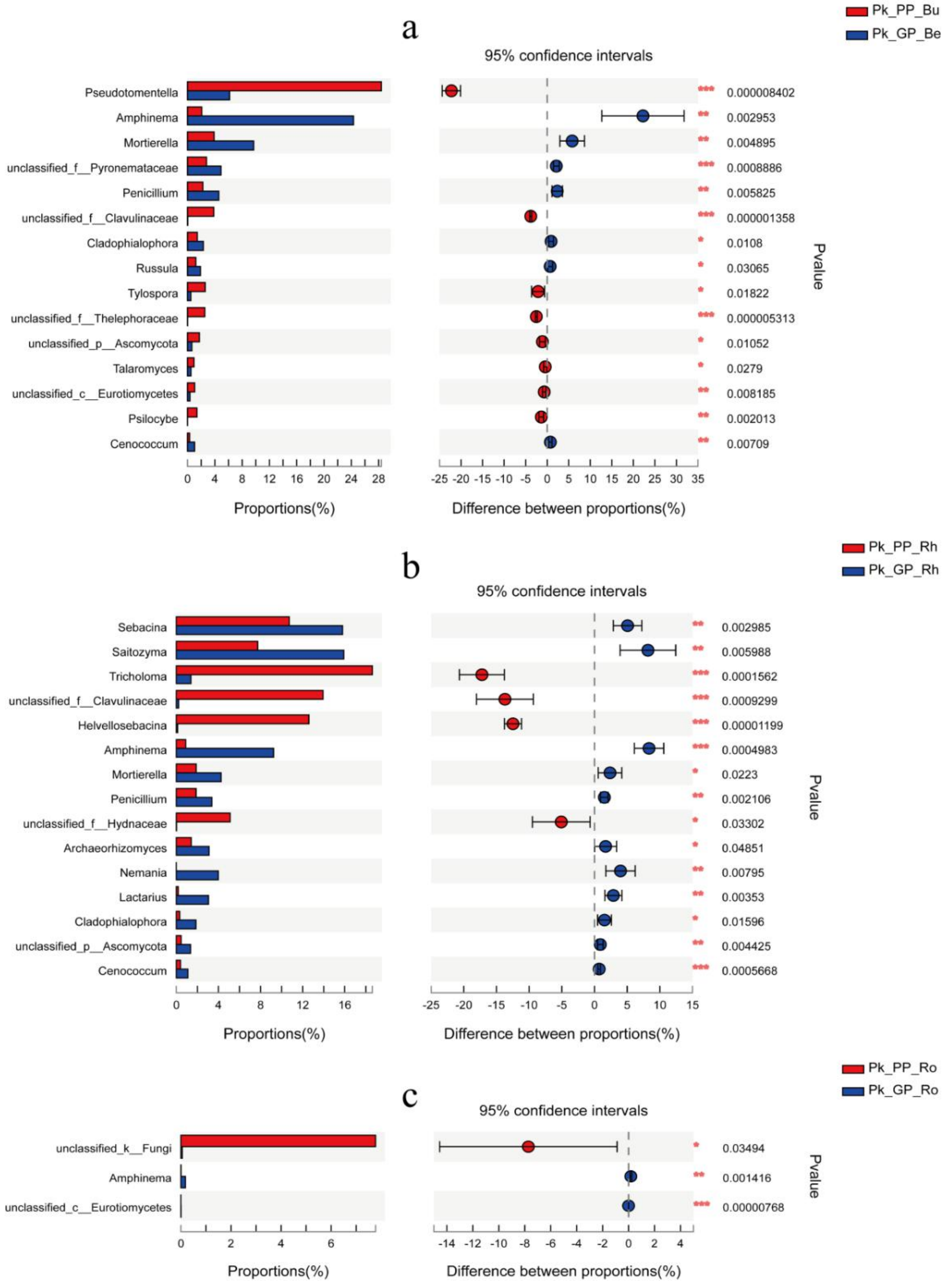

Supplementary Figure 6. Student t-test bar plots for different compartments of fungal community of *P.k.* on genus level. PP, pure pine forest, GP, agroforestry system.

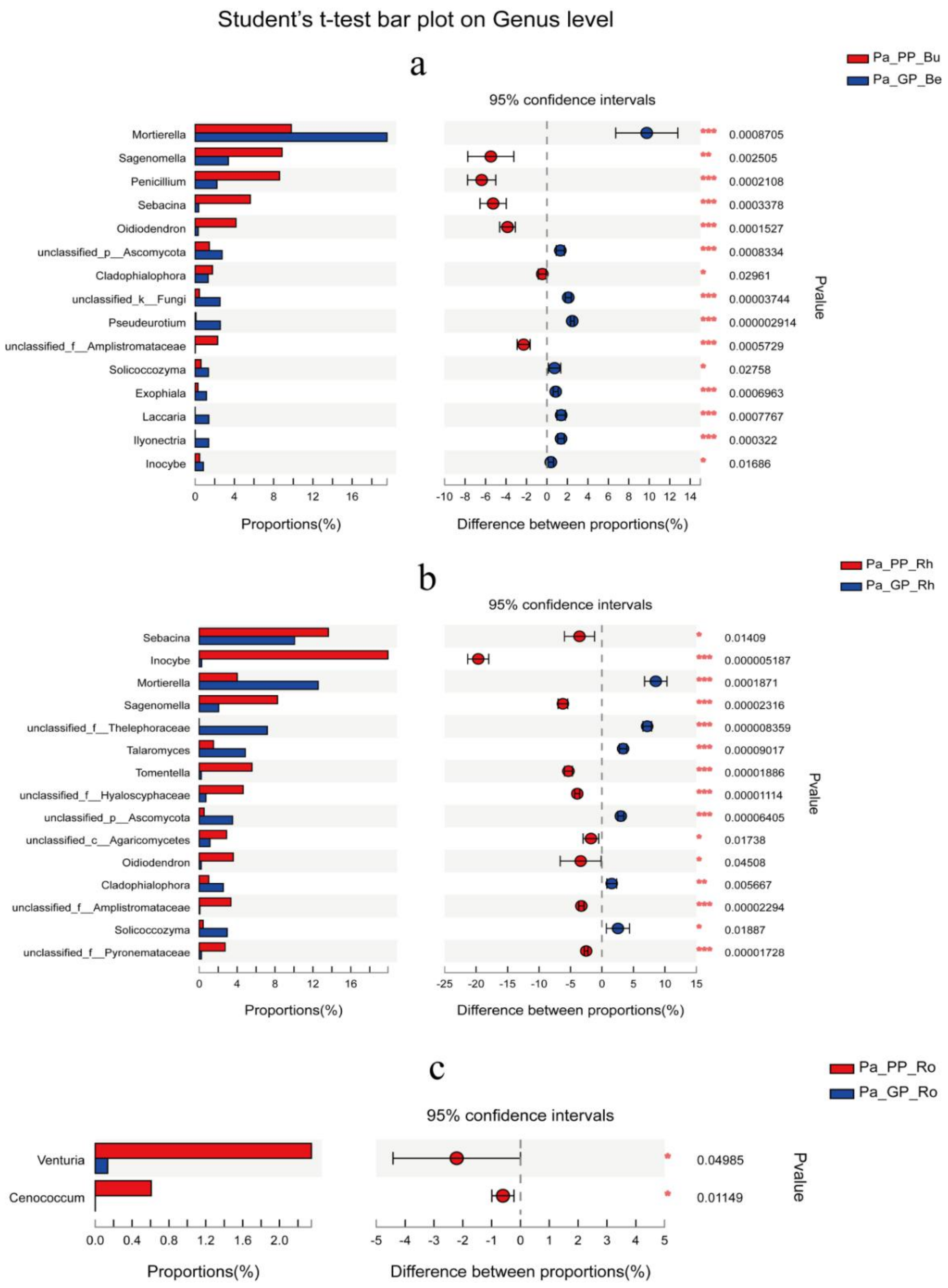

Supplementary Figure 7. Student t-test bar plots for different compartments of fungal community of *P.a.* on genus level. PP, pure pine forest, GP, agroforestry

system.

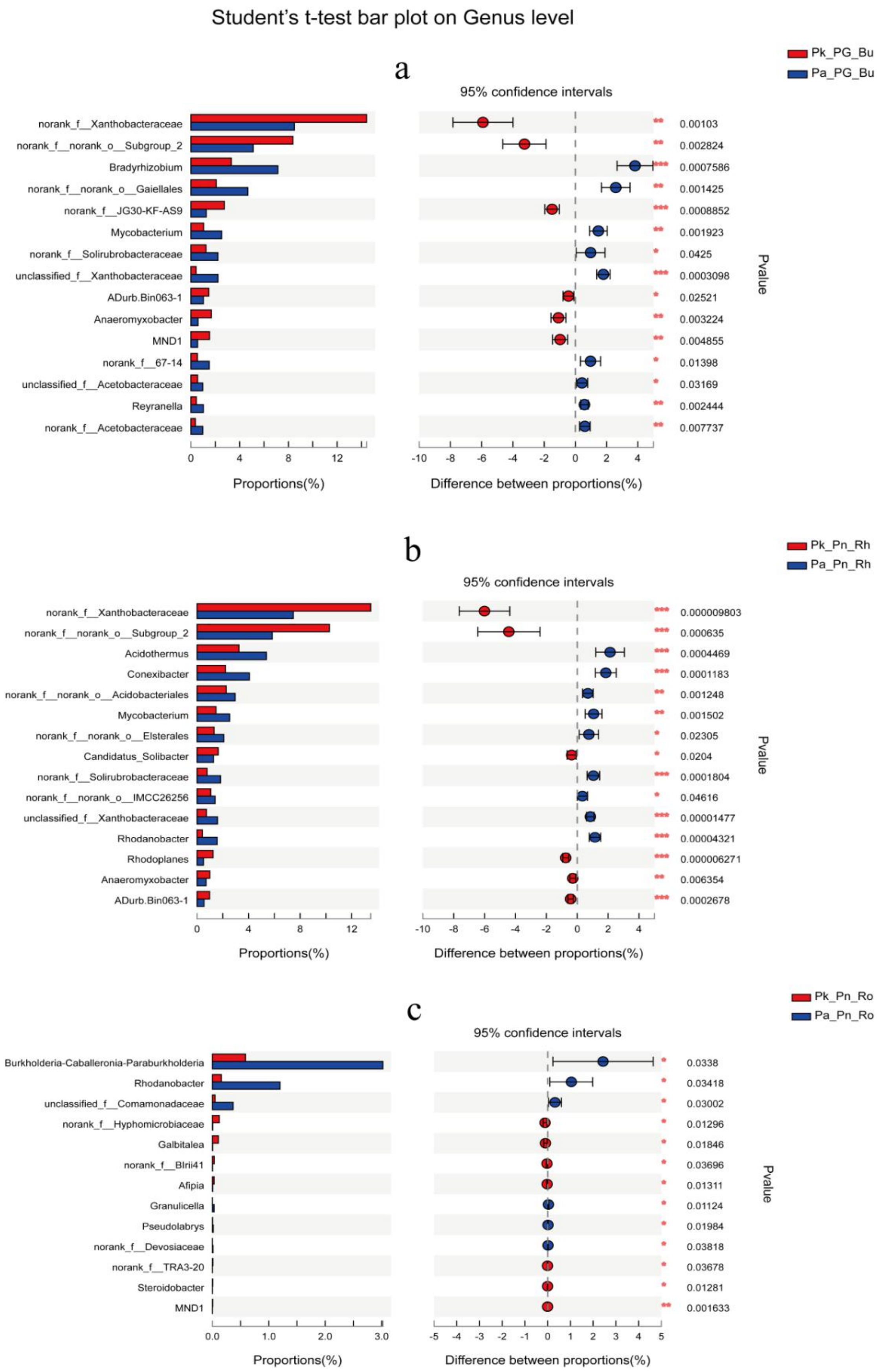
Student's t-test bar plot on Genus level
a
Pk_PG_Bu
Pa_PG_Bu
95% confidence intervals
norank_f__Xanthobacteraceae
norank_f__norank_o__Subgroup_2
Bradyrhizobium
norank_f__norank_o__Gaiellales
norank_f__JG30-KF-AS9
Mycobacterium
norank_f__Solirubrobacteraceae
unclassified_f__Xanthobacteraceae
ADurb.Bin063-1
Anaeromyxobacter
MND1
norank_f__67-14
unclassified_f__Acetobacteraceae
Reyranella
norank_f__Acetobacteraceae
0.00103
0.002824
0.0007586
0.001425
0.0008852
0.001923
0.0425
0.0003098
0.02521
0.003224
0.004855
0.01398
0.03169
0.002444
0.007737
Pvalue
Proportions(%)
Difference between proportions(%)
b
Pk_Pn_Rh
Pa_Pn_Rh
95% confidence intervals
norank_f__Xanthobacteraceae
norank_f__norank_o__Subgroup_2
Acidothermus
Conexibacter
norank_f__norank_o__Acidobacteriales
Mycobacterium
norank_f__norank_o__Elsterales
Candidatus_Solibacter
norank_f__Solirubrobacteraceae
norank_f__norank_o__IMCC26256
unclassified_f__Xanthobacteraceae
Rhodanobacter
Rhodoplanes
Anaeromyxobacter
ADurb.Bin063-1
0.000009803
0.000635
0.0004469
0.0001183
0.001248
0.001502
0.02305
0.0204
0.0001804
0.04616
0.00001477
0.00004321
0.000006271
0.006354
0.0002678
Pvalue
Proportions(%)
Difference between proportions(%)
c
Pk_Pn_Ro
Pa_Pn_Ro
95% confidence intervals
Burkholderia-Caballeronia-Paraburkholderia
Rhodanobacter
unclassified_f__Comamonadaceae
norank_f__Hyphomicrobiaceae
Galbitalea
norank_f__Blrii41
Afipia
Granulicella
Pseudolabrys
norank_f__Devosiaceae
norank_f__TRA3-20
Steroidobacter
MND1
0.0338
0.03418
0.03002
0.01296
0.01846
0.03696
0.01311
0.01124
0.01984
0.03818
0.03678
0.01281
0.001633
Pvalue
Proportions(%)
Difference between proportions(%)

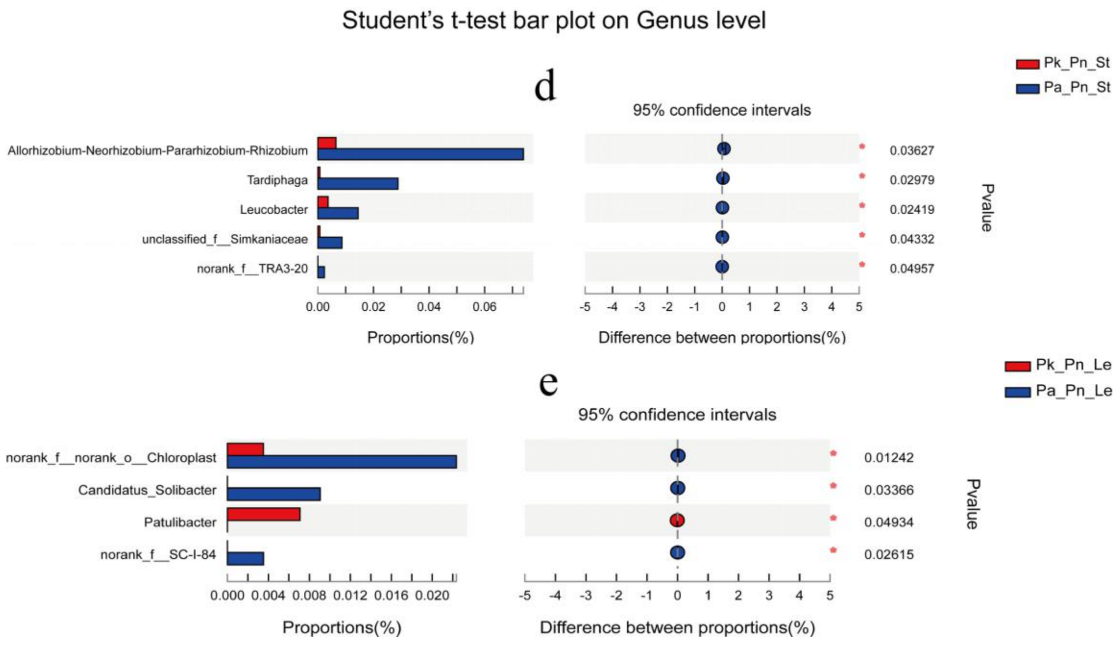

Supplementary Figure 8. Student t-test bar plots for different compartments of fungal community of *P.n.* on genus level. Pk, *P.n.-P.k.* agroforestry system, Pa, *P.n.-P.a.* agroforestry system.

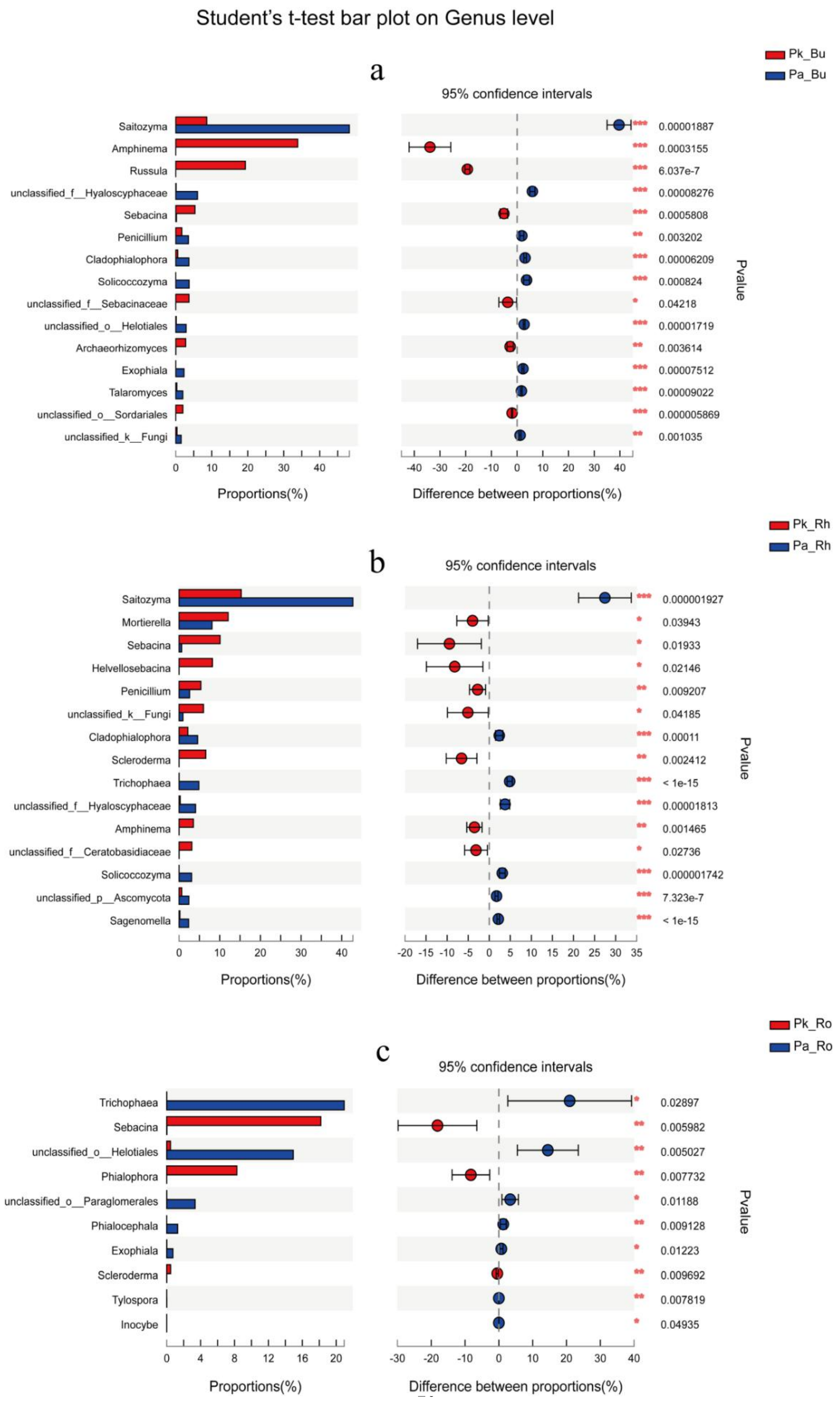
Student's t-test bar plot on Genus level
a
Pk_Bu
Pa_Bu
95% confidence intervals
Saitozyma
Amphinema
Russula
unclassified_f__Hyaloscyphaceae
Sebacina
Penicillium
Cladophialophora
Solicoccozyma
unclassified_f__Sebacinaceae
unclassified_o__Helotiales
Archaeorhizomyces
Exophiala
Talaromyces
unclassified_o__Sordariales
unclassified_k__Fungi
0.00001887
0.0003155
6.037e-7
0.00008276
0.0005808
0.003202
0.00006209
0.000824
0.04218
0.00001719
0.003614
0.00007512
0.00009022
0.000005869
0.001035
Pvalue
Proportions(%)
Difference between proportions(%)
b
Pk_Rh
Pa_Rh
95% confidence intervals
Saitozyma
Mortierella
Sebacina
Helvellosebacina
Penicillium
unclassified_k__Fungi
Cladophialophora
Scleroderma
Trichophaea
unclassified_f__Hyaloscyphaceae
Amphinema
unclassified_f__Ceratobasidiaceae
Solicoccozyma
unclassified_p__Ascomycota
Sagenomella
0.000001927
0.03943
0.01933
0.02146
0.009207
0.04185
0.00011
0.002412
< 1e-15
0.00001813
0.001465
0.02736
0.000001742
7.323e-7
< 1e-15
Pvalue
Proportions(%)
Difference between proportions(%)
c
Pk_Ro
Pa_Ro
95% confidence intervals
Trichophaea
Sebacina
unclassified_o__Helotiales
Phialophora
unclassified_o__Paraglomerales
Phialocephala
Exophiala
Scleroderma
Tylospora
Inocybe
0.02897
0.005982
0.005027
0.007732
0.01188
0.009128
0.01223
0.009692
0.007819
0.04935
Pvalue
Proportions(%)
Difference between proportions(%)

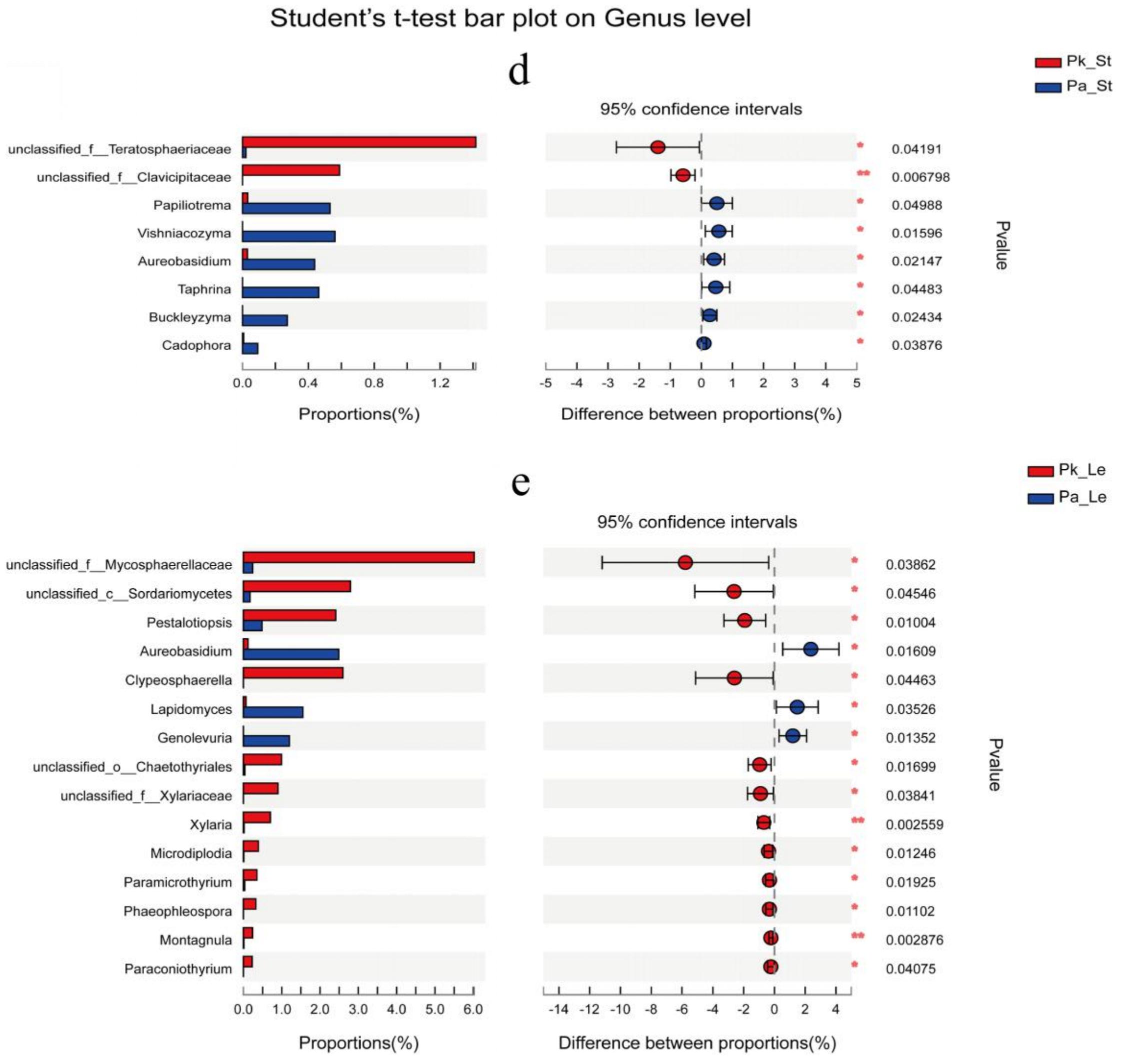

Supplementary Figure 9. Student t-test bar plots for different compartments of fungal community of *P.n.* on genus level. Pk, *P.n.-P.k.* agroforestry system, Pa, *P.n.-P.a.* agroforestry system.
